# Synthesis of dibenzoarsole derivatives from biarylborates *via* the twofold formation of C–As bonds using arsenium dication equivalents

**DOI:** 10.1039/d5sc05528h

**Published:** 2025-10-10

**Authors:** Kazutoshi Nishimura, Hiroki Iwamoto, Yuji Nishii, Koji Hirano

**Affiliations:** a Department of Applied Chemistry, Graduate School of Engineering, Osaka University Suita Osaka 565-0871 Japan k_hirano@chem.eng.osaka-u.ac.jp; b Innovative Catalysis Science Division, Institute for Open and Transdisciplinary Research Initiatives (ICS-OTRI), Osaka University Suita Osaka 565-0871 Japan

## Abstract

A strategy for the generation of arsenium dication equivalents from readily available and easy-to-handle phenylarsine oxide and Tf_2_O has been developed. The *in situ*-generated dication equivalent can react with biarylborates to directly produce the corresponding dibenzoarsoles, which are difficult to prepare by other means, *via* the successive formation of inter- and intramolecular C–As bonds. Furthermore, the unique oxygen atom insertion into the C–As bond in the dibenzoarsole is developed to form the corresponding [1,2]oxarsinine derivative.

## Abstract 2

A strategy for generation of As dication equivalents from PhAs=O and Tf_2_O has been developed to dircetly convert biarylborates into the corresponding dibenzoarsoles. Furthermore, skeletal edditing of obtained benzoarsoles is described.

## Graphical abstract

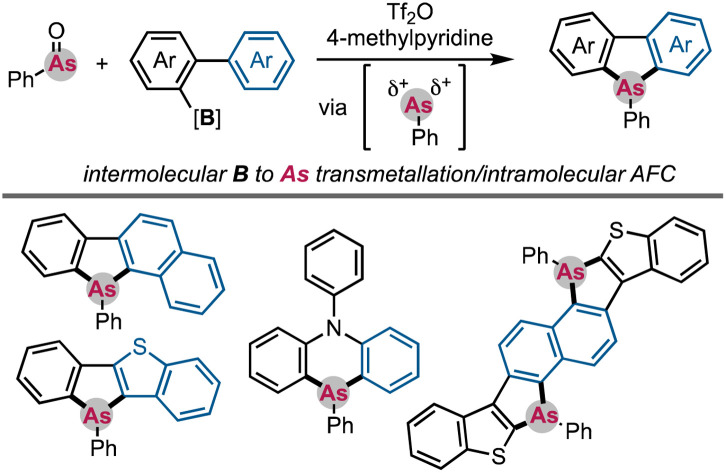

## Introduction

The design and synthesis of π-conjugated molecules are of significant interest for their applications in organic electronics, photovoltaics, and light-emitting diodes.^1^ Enhancing the properties of these systems is often achieved by incorporating heteroatoms such as nitrogen, sulfur, and oxygen into the π-conjugated structure, which significantly alters their electronic distribution, optical properties, and molecular stability.^2^

Although a variety of heterocyclic compounds have already been synthesized, the development of arsenic-containing heterocyclic compounds still remains relatively limited. Compared to the traditional strategies with highly toxic and volatile arsenic chlorides or H-arsines,^3^ their synthesis has witnessed remarkable progress as a result of appearance and design of reagents, catalysis, and conditions for the C–As bond forming reaction.^4^ However, the electrophilic C–As bond forming reaction is particularly restricted in scope and generality. As one of the breakthroughs, Naka and Imoto have recently proposed a transformation that is based on a non-volatile intermediate that is *in situ* generated PhAsI_2_, which in turn can be obtained from the non-toxic and solid (PhAs)_6_.^5^ While this protocol eliminates the use of hazardous arsenic precursors, the lower electrophilicity of PhAsI_2_ still requires highly reactive organometallic reagents, such as Grignard or lithium reagents for the formation of C–As bonds. Therefore, the development of efficient and concise reactions that produce C–As bonds, particularly for the synthesis of As-containing aromatic compounds, from stable and less toxic starting materials with high functional-group compatibility would be highly desirable.

**Scheme 1:**
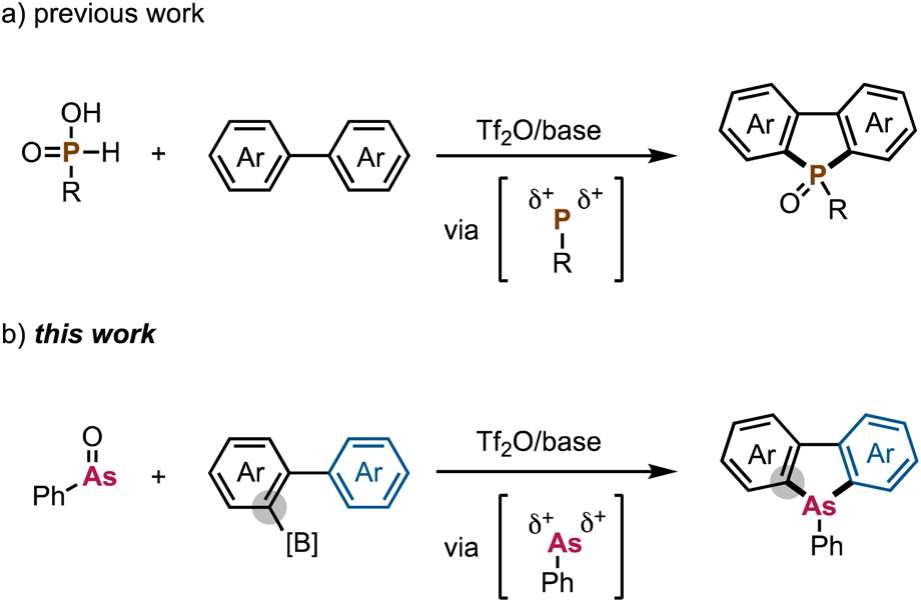
(a) Direct synthesis of dibenzophospholes from simple biaryls *via* P dication equivalents and (b) synthesis of dibenzoarsoles from biarylborates *via* the twofold formation of C–As bonds using arsenium dication equivalents.

In one of our previous reports, we have already used phosphenium dication equivalents to efficiently synthesize dibenzophospholes from simple biaryls and phosphinic acids (Scheme 1a).^6^ Inspired by this success, we anticipated that this strategy could potentially be expanded to the generation and use of arsenium dication equivalents,^7^ which can be obtained from solid and non-volatile Ph-As=O^8^ and Tf_2_O. Here, we report a straightforward synthetic route to dibenzoarsole derivatives from biarylborates *via* arsenium dication equivalents, which mediate the twofold formation of C–As bonds (Scheme 1b). This newly developed protocol enables the concise synthesis of dibenzoarsoles without the need to employ highly reactive organometallic reagents and/or dangerous arsenic precursors. The biarylborate can be readily and modularly prepared by the Suzuki–Miyaura coupling/Miyaura boration sequence from the readily available starting substrates.^9^ Moreover, this protocol can also be applied to the synthesis of six-membered arsacycles and a largely π-extended dibenzoarsole derivative. Furthermore, we observed that dibenzoarsole oxides undergo ring-expansion reactions when treated with *m*CPBA, which leads to oxygen-inserted arsenic-containing heterocycles. We also note that during the course of this study Szewczyk, Sobolewski, Gryko and coworkers reported a related intramolecular electrophilic C–As bond forming reaction of triarylarsine oxide under Tf_2_O-promoted conditions, delivering the π-extended arsolium salt, but the synthesis of neutral arsine derivatives still remains a challenge.^10^

## Results and discussion

**Scheme 2:**
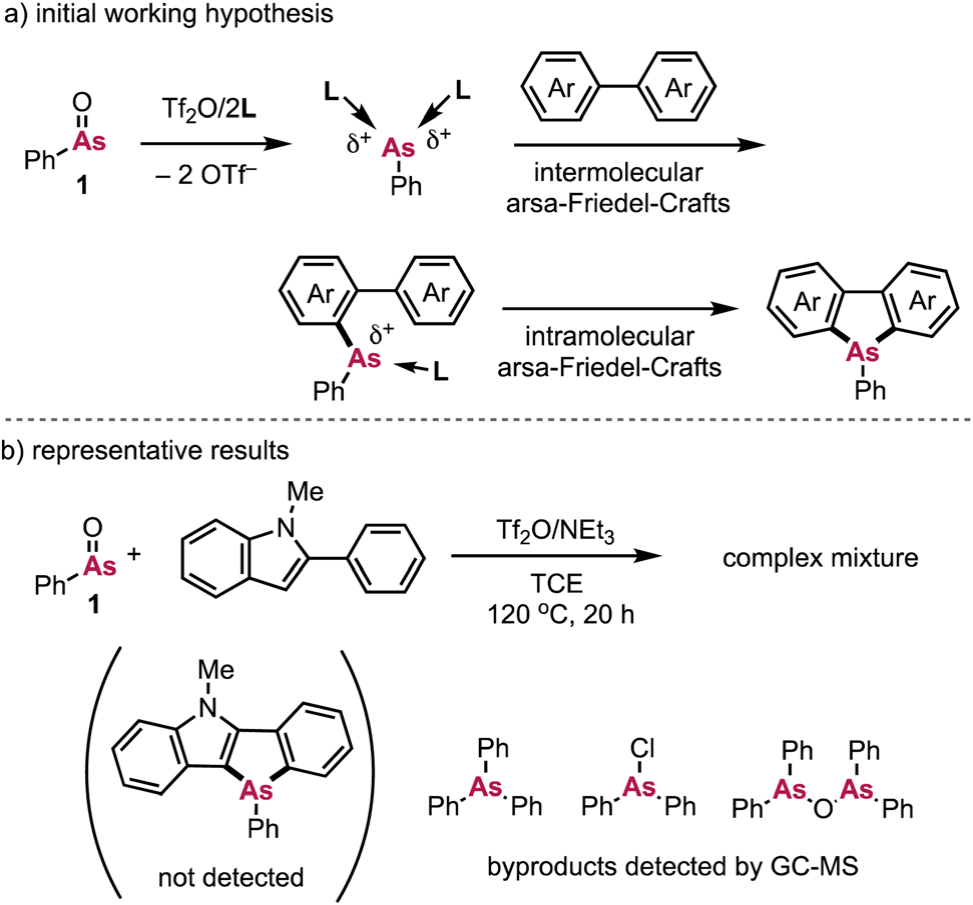
(a) Our initial working hypothesis for the direct synthesis of dibenzoarsoles from simple biaryls using arsenium dication equivalents and (b) representative unsuccessful results; L = Lewis base.

**Scheme 3:**
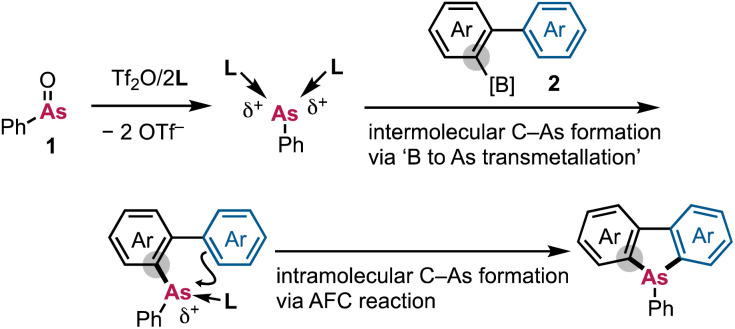
Revised working hypothesis for the synthesis of dibenzoarsole derivatives from biarylborates 2*via* the twofold formation of C–As bonds using arsenium dication equivalents.

Based on our previously reported synthesis of dibenzophospholes mediated by phosphenium dication equivalents, we initially tried the reaction of some simple biaryls such as *N*-methyl-2-phenylindole with PhAs=O (1) and Tf_2_O. Initially, our working hypothesis consisted of (1) the generation of highly electrophilic, coordinatively unsaturated arsenic dication equivalents upon treatment of 1 with Tf_2_O,^11^ whereby the two OTf ligands are displaced by external neutral Lewis bases (L), (2) an intermolecular arsa-Friedel–Crafts (AFC)-type reaction, and (3) a ring-closing reaction *via* an intramolecular AFC reaction (Scheme 2a).^7*c*^ However, as far as we tested, this approach did not yield the desired dibenzoarsole product (Scheme 2b). Instead, Ph_3_As, Ph_2_AsCl,^12^ and (Ph_2_As)_2_O were detected by GC-MS analysis, which suggests that the cationic arsenic species is generated *in situ*, and that ligand scrambling on the cationic As moiety^13^ is faster than the desired intermolecular formation of the C–As bond with the simple biaryl. Consequently, we hypothesized that more nucleophilic substrates, such as biarylboronic acids (2) could potentially be more effective to promote the formation of the first, intermolecular C–As bond, which would render the formation of the second, intramolecular C–As bond feasible.^7*c*^ This modified working hypothesis is illustrated in Scheme 3.

In our updated working hypothesis, the highly electrophilic arsenium dication equivalent is generated from PhAs=O (1), Tf_2_O, and L. The formation of the first C–As bond is considered to occur between the cationic As moiety and the arylboronic acid (B-to-As transmetallation), followed by an intramolecular AFC reaction that forms the second C–As bond.

**Scheme 4:**
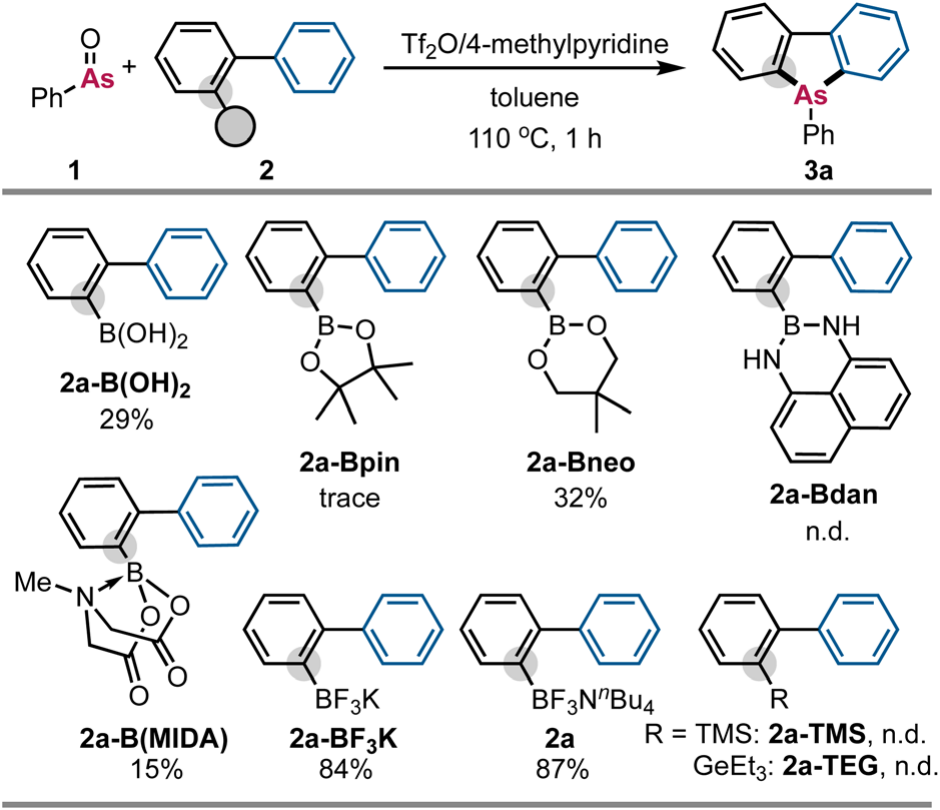
Scope of biphenylboron derivatives. The NMR yields of the desired target (3a) are shown. Reaction conditions: 1 (0.24 mmol), 2 (0.10 mmol), Tf_2_O (0.24 mmol), 4-methylpyridine (0.36 mmol), toluene (1.5 mL), 110 °C, 1 h, N_2_. n.d. = not detected.

When we treated PhAs=O (1; 0.24 mmol) with biphenylboronic acid (2a-B(OH)_2_; 0.1 mmol), Tf_2_O (0.24 mmol), and 4-methylpyridine (0.36 mmol) in toluene at 110 °C for 1 hour, we observed the formation of the corresponding dibenzoarsole (3a)^5*a*^ in 29% NMR yield (Scheme 4). These preliminary intriguing results prompted us to explore other biphenylboron derivatives. However, none of our attempts to use biphenylboronic acid pinacol ester (2a-Bpin), biphenylboronic acid neopentylglycol ester (2a-Bneo), biphenyl(naphthalene-1,8-diamino)boron (2a-Bdan), and biphenyl MIDA boronate (2a-B(MIDA)) improved the reaction efficiency. On the other hand, we found that biaryl trifluoroborates (2a-BF_3_K and 2a) gave better results. In particular, ammonium borate 2a afforded much better results owing to its high solubility. In contrast, the desired product (3a) was not obtained when TMS- and Et_3_Ge-substituted biphenyls (2a-TMS and 2a-TEG) were used. Several observations that we made during our optimization studies should be noted here. We also examined several bases other than 4-methylpyridine, but no improvement was observed. Any other dehydrating agents such as (CF_3_CO)_2_O, Ts_2_O, Ac_2_O, and PhNTf_2_, did not promote the reaction. The much better leaving ability of TfO^−^ is believed to be critical for successful sequential C–As bond formation while PhNTf_2_ cannot form the arsenic dication equivalent because of its lower electrophilicity than that of Tf_2_O. PhAs=O (1) is an indispensable arsenic source in this reaction, and 3a and/or its oxide (4a) was formed only in <10% yield from Ph_3_As or PhAs(O)(OH)_2_ instead of 1 under otherwise identical conditions (for details, see the SI).

**Scheme 5:**
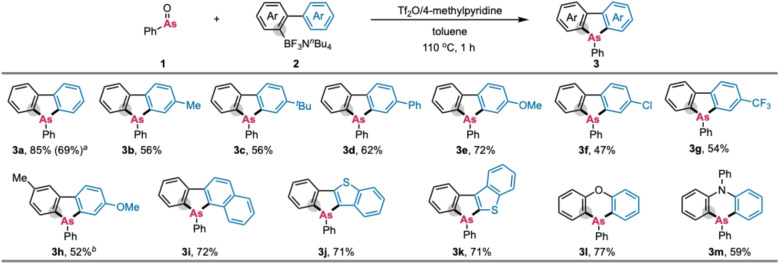
Synthesis of dibenzoarsoles 3*via* the twofold formation of C–As bonds between biarylborates 2 and phenylarsine oxide (1). Reaction conditions: 1 (0.24 mmol), 2 (0.10 mmol), Tf_2_O (0.24 mmol), 4-methylpyridine (0.36 mmol), toluene (1.5 mL), 110 °C, 1 h, N_2_. Isolated yields are shown. ^*a*^On the 1.0 mmol scale. ^*b*^With Et_3_N instead of 4-methylpyridine at 60 °C.

With the optimal conditions established, we investigated the scope of ammonium borates (2) with versatile biaryl skeletons (Scheme 5). The standard reaction conditions proved equally compatible with electron-neutral (Me, *t*-Bu, Ph), -donating (OMe), and -withdrawing (Cl and CF_3_) groups, resulting in the formation of the corresponding dibenzoarsoles (3b–g) in good yield (47–72%). The substituent on the BF_3_-substituted left ring was also tolerated (3h), where the electron-donating Me group facilitated the reaction even at lower temperature (60 °C). This reactivity trend is consistent with the intermolecular transmetallation mechanism in the first C–As formation (Scheme 3). Additionally, substrates with a higher π-conjugated system (3i) and heterocyclic benzothiophenes (3j^5*g*^ and 3k) also underwent the reaction smoothly. This strategy was further extended to the synthesis of six-membered arsacycles. Borates containing diaryl ether and triarylamine moieties were directly converted to the corresponding six-membered phenoxarsine 3l^5*h*^ and phenoarsazine 3m^14^ in acceptable yield. The reaction could also be performed on a 10-fold increased scale (3a), which showcases the practical utility and good reproducibility of the process. As a general trend, the more electron-rich aromatic rings (3a–e) showed higher reactivity than the electron-deficient ones (3f and g). In addition, the naphthalene ring selectively reacted at the more congested but more electron-rich *α* position (3i). These features are consistent with the aromatic electrophilic substitution mechanism, that is, AFC-type reaction in the second C–As bond formation process as proposed in Scheme 3.

**Scheme 6:**
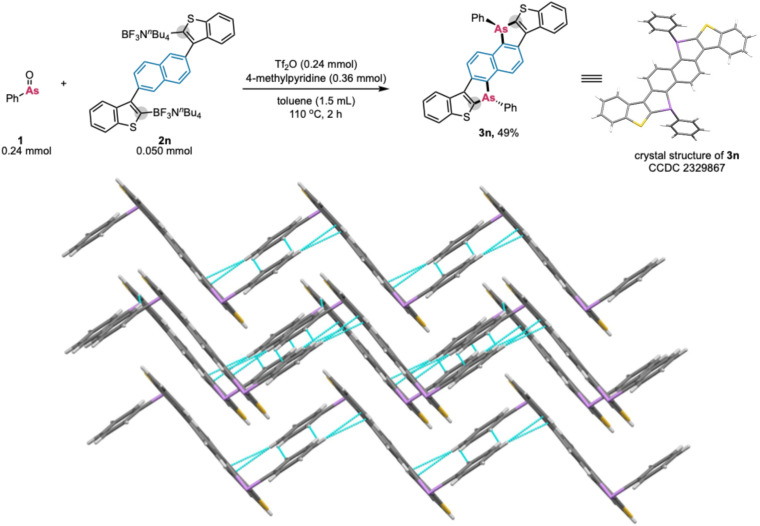
Synthesis of S,As-acene (3n) *via* the fourfold formation of C–As bonds and its crystal structure.

We next attempted the synthesis of a largely π-extended dibenzoarsole derivative. 2,6-Bis(3-benzothienylborate)naphthalene 2n was transformed, *via* the fourfold formation of C–As bonds, to the corresponding highly condensed, bent-type S,As-acene 3n in 49% yield (Scheme 6). The solid-state structure of 3n was unambiguously confirmed by single-crystal X-ray diffraction analysis (CCDC 2329867). The single crystal of 3n showed face-to-face slipped columnar structure, where the stacking distance was relatively long (*ca.* 3.839 Å). This result indicates the weak π–π stacking because of the intermolecular steric repulsions arising from the two Ph rings on arsenic atoms. It is also noteworthy that no any special interactions including arsenic and sulfur atoms were observed while there were some CH/CH and CH/π interactions between the Ph ring on arsenic and the edge of benzothiophene moiety.

**Fig. 1:**
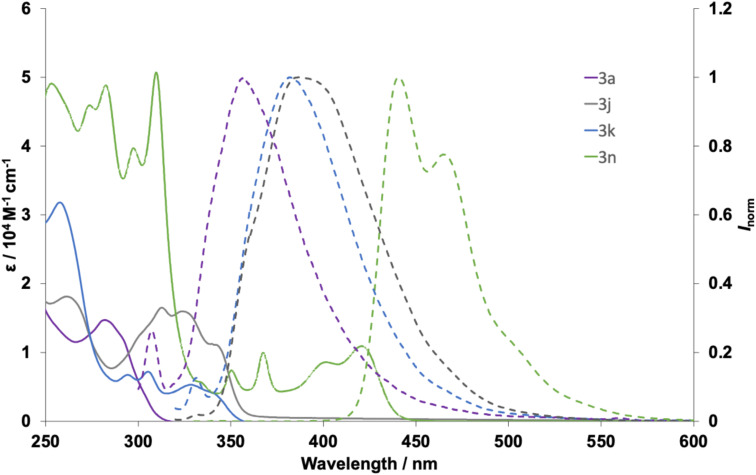
UV/vis absorption (solid lines) and fluorescence spectra (dashed lines) of 3a, 3j, 3k, and 3n in CHCl_3_ (1.0 × 10^−5^ M).

The preliminary photoluminescent properties of 3a, 3j, 3k, and 3n were investigated. The compounds 3a^5*a*^ and 3j^5*g*^ were reported in the literature, but their properties were also surveyed again to compare those of regioisomeric 3k and more π-extended benzothiophene-fused derivative 3n. Their UV/vis absorption and fluorescence spectra in CHCl_3_ (1.0 × 10^−5^ M) are summarized in Fig. 1 and Table 1. All compounds exhibited little to no fluorescence, compared to the N-, O-, and S-analogues (carbazole, dibenzofuran, and dibenzothiophene, respectively). This is most likely due to the presence of arsenic, a heavy atom, which promotes intersystem crossing to the triplet state, thus quenching fluorescence emission. This is a kind of typical heavy atom effects owing to the spin–orbital interaction, suggesting the possibility for applications as unique phosphorescence materials. The higher-fused bisbenzothiophene derivative 3n showed absorption peaks in the visible region.

**Table 1:** Optical properties of 3a, 3j, 3k, and 3n^a^

3	*λ*_abs_ (nm) (*ε* (10^4^ M^−1^ cm^−1^))	*λ*_Fl_ b (nm)	*Φ* (%)
3a	282 (1.5)	307, 357	1
3j	262 (1.8), 313 (1.6), 324 (1.6)	387	1
3k	258 (3.2), 294 (0.67), 305 (0.72), 328 (0.53)	331, 382	1
3n	253 (4.9), 274 (4.6), 282 (4.9), 297 (4.0), 310 (5.0), 350 (0.74), 367 (1.0), 402 (0.86), 421 (1.1)	441, 465	3

a Measured in CHCl_3_ (1.0 × 10^−5^ M).

b Excited at 280 (3a), 300 (3j), 300 (3k), and 310 nm (3n), respectively.

The electrochemical properties of the aforementioned compounds were examined by cyclic voltammetry (CV) and differential pulse voltammetry (DPV) in *o*-dichlorobenzene/MeCN (10/1, v/v, for 3a, 3j, and 3k) or dichloromethane (for 3n) with tetrabutylammonium hexafluorophosphate (Bu_4_NPF_6_) as the supporting electrolyte *versus* ferrocene/ferrocenium ion (Fc/Fc^+^) (Fig. S3–6), and their HOMO and LUMO levels were estimated according to the first oxidation potentials and the optical band gaps (*E*^opt^_g_) (Table 2). The cyclic voltammograms of 3a, 3j, 3k, and 3n showed irreversible oxidation waves, and the oxidation potential values *E*_ox_^1/2^ were thus determined by DPV. In comparison with the parent dibenzoarsole 3a, the benzothiophene-fused dibenzoarsole derivatives (3j, 3k, and 3n) exhibited *E*_ox_^1/2^ values that were shifted in negative direction probably due to the presence of the electron-donating thiophene ring. Given its lower LUMO and higher HOMO levels, a larger intramolecular charge-transfer ability is suggested for 3n. Almost all the aforementioned values are identical for 3j and 3k, which suggests that the orientation of the benzothiophene ring fusion does not significantly affect the optoelectronic properties.

**Table 2:** Absorption wavelengths, HOMO–LUMO energy gaps, and DPV data of 3a, 3j, 3k, and 3n

3	a *λ* ^abs^_onset_ (nm)	b *E* ^opt^_g_ (eV)	c *E*_ox_^1/2^ (V)	d *E*_HOMO_ (eV)	e *E*_LUMO_ (eV)
3a	308	4.03	1.35	−6.15	−2.12
3j	357	3.47	0.96	−5.76	−2.29
3k	356	3.48	0.90	−5.70	−2.22
3n	441	2.81	0.67	−5.47	−2.66

a Measured in CHCl_3_.

b Determined from the onset of the normalized absorption spectra.

c Performed in *o*-dichlorobenzene/MeCN (10 : 1, v/v for 3a, 3j, and 3k) or CH_2_Cl_2_ (for 3n) in the presence of Bu_4_NPF_6_. *v* = 0.10 V s^−1^ (3a), 0.050 V s^−1^ (3j and 3k), and 0.030 V s^−1^ (3n), *versus* Fc/Fc^+^.

d The approximation for Fc/Fc^+^ level is −4.8 eV *versus* vacuum: *E*_HOMO_ = −4.8 − *E*_ox_^1/2^.

e Estimated from *E*_HOMO_ and *E*^opt^_g_: *E*_LUMO_ = *E*_HOMO_ + *E*^opt^_g_.

**Scheme 7:**
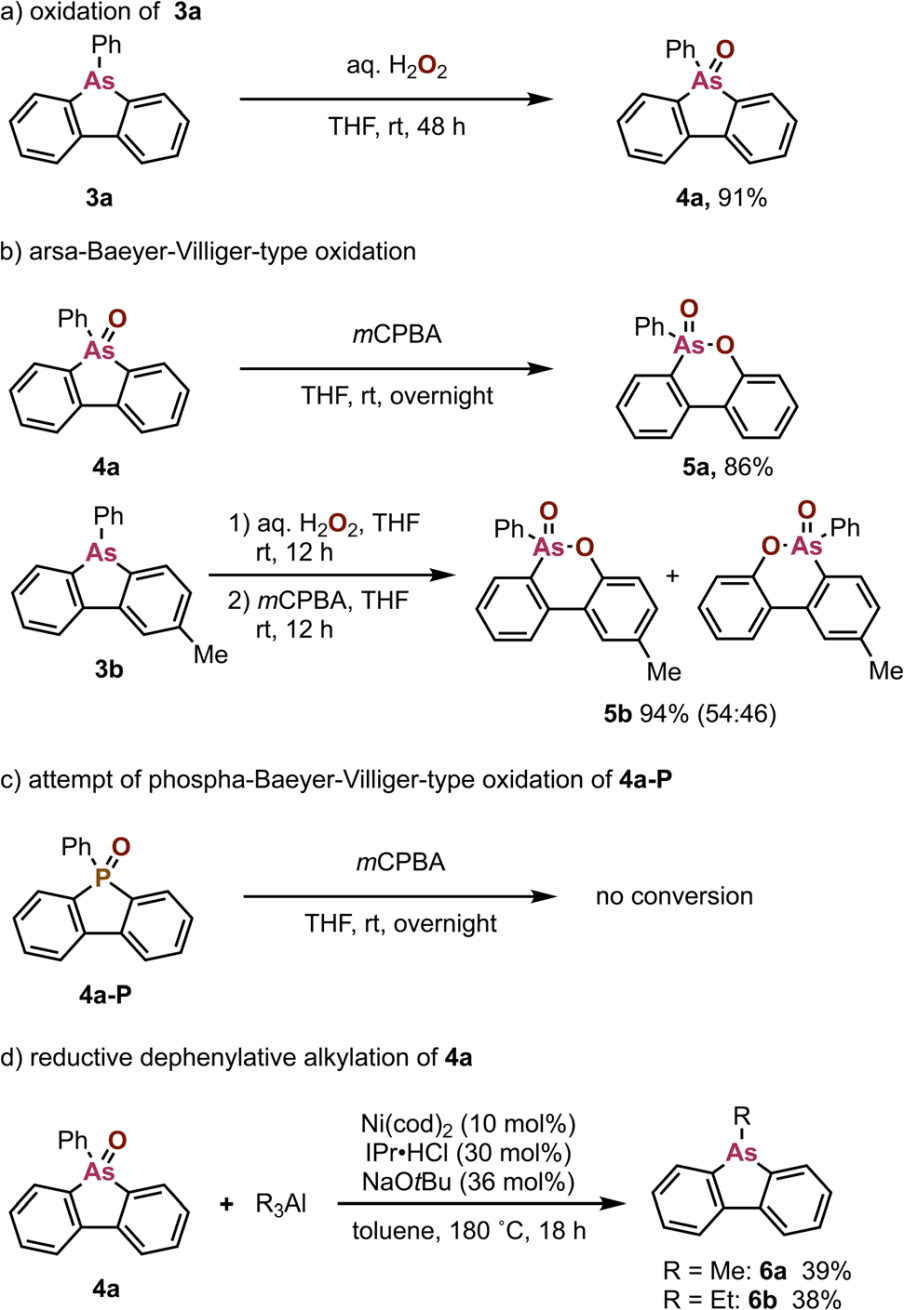
(a) Oxidation of 3a, (b) arsa-Baeyer–Villiger-type oxidation of 4a and 3b, (c) attempted phospha-Baeyer–Villiger-type oxidation of4a-P, and (d) reductive dephenylative alkylation of 4a. For detailed reaction conditions, see the SI.

Finally, we explored the derivatization of the obtained dibenzoarsoles. Dibenzoarsole 3a was successfully oxidized with aqueous hydrogen peroxide to furnish dibenzoarsole oxide 4a^15^ in 91% yield (Scheme 7a). The reaction of 4a with *m*CPBA promoted an arsa-Baeyer–Villiger oxidation to produce a six-membered arsenic-containing cyclic compound (5a) *via* an oxygen-atom insertion into the C–As bond (Scheme 7b). The methyl-substituted 3b could also be converted to the corresponding ring-expanded product 5b by sequential treatment with hydrogen peroxide and *m*CPBA albeit with poor regioselectivity. It should be noted here that examples of oxygen-insertion reactions into aromatic heterocyclic compounds are scarce.^16^ Indeed, the phosphorus analogue, *i.e.*, benzophosphole oxide 4a-P, was not converted under otherwise identical conditions (Scheme 7c), which highlights the unique reactivity of dibenzoarsole oxides. These ring-expansion reactions represent a kind of skeletal editing of heteroaromatics,^17^ which enables access to novel arsenic-containing cyclic compounds. Furthermore, dibenzoarsole oxide 4a reacted with trialkylaluminums under Ni catalysis^18^ to produce the corresponding 5-alkyldibenzoarsoles 6a^5*a*^ and 6b with the removal of Ph group and oxygen (Scheme 7d), which can complement inaccessibility to the toxic methyl- and ethylarsine oxide starting substrates with high volatility.

## Conclusions

We have developed a new strategy for generation of arsenium dication equivalents from stable, easy-to-handle, and relatively benign phenylarsine oxide and Tf_2_O. The *in situ*-generated dication equivalent can react with biarylborates to produce the corresponding dibenzoarsoles *via* the successive formation of inter- and intramolecular C–As bonds. This protocol enables the synthesis of oxygen- and nitrogen-containing arsenic six-membered-ring derivatives as well as a highly condensed benzothiophene-containing dibenzoarsole. Moreover, we have investigated the oxygen atom insertion into the As–C bond in the dibenzoarsole oxide to obtain the [1,2]oxarsinine heterocycle. The most salient feature of this method is that it is based on the non-volatile phenylarsine oxide and diarylboronic acids; both are stable and of mild reactivity. This protocol thus provides an avenue to a variety of arsenic-containing heterocycles that have previously been difficult to access, as exemplified by largely π-extended octacyclic system 3n bearing two arsole and two thiophene rings in the molecular core.

## Author contributions

K. N. and K. H. conceived the idea. K. N. and H. I. performed all experiments. Y. N. assisted X-ray analysis. K. H. supervised the project. K. N. and K. H. wrote the manuscript. All the authors discussed the results and commented on the manuscript.

## Conflicts of interest

There are no conflicts to declare.

## Supplementary Material

SC-016-D5SC05528H-s001

SC-016-D5SC05528H-s002

## Data Availability

CCDC 2329867 contains the supplementary crystallographic data for this paper.^19^ All experimental procedures and spectroscopic data can be found in the supplementary information (SI). Supplementary information is available. See DOI: https://doi.org/10.1039/d5sc05528h.
