# Supplementary material for: Synthesis of dibenzoarsole derivatives from biarylborates via the twofold formation of C–As bonds using arsenium dication equivalents
Source: Chem Sci. 2025 Oct 10;16(45):21433–9. doi: 10.1039/d5sc05528h (PMC12522149; doi:10.1039/d5sc05528h)

Supplementary Information (SI) for Chemical Science.
This journal is © The Royal Society of Chemistry 2025

Supplementary Information

Synthesis of dibenzoarsole derivatives from biarylborates by double C–As bond formation via arsenium dication equivalents

Kazutoshi Nishimura,† Hiroki Iwamoto,† Yuji Nishii†, ‡ and Koji Hirano*,†, ‡

†*Department of Applied Chemistry, Graduate School of Engineering, Osaka University, Suita, Osaka 565-0871, Japan*

‡*Innovative Catalysis Science Division, Institute for Open and Transdisciplinary Research Initiatives (ICS-OTRI), Osaka University, Suita, Osaka 565-0871, Japan*

k_hirano@chem.eng.osaka-u.ac.jp

Contents

**Instrumentation and Chemicals**	S1–S2
**Experimental Procedures and Characterization Data**	S3–S10
**Detailed Optimization Studies**	S11–S12
**X-Ray Analysis**	S13
**Photoluminescence Properties**	S14
**Electrochemical Properties**	S15–S17
**References**	S18
**Copies of NMR Charts**	S19–S38

Instrumentation and Chemicals

^{1}H, ^{13}C{^{1}H}, and ^{19}F{^{1}H} spectra were recorded at 400 MHz, 100 MHz, and 162 MHz, respectively, for $CDCl_3$ or CD_2Cl_2 solutions. HRMS data were obtained by APCI using a TOF. GC analysis was carried out using a silicon OV-17 column (i. d. 2.6 mm x 1.5 m) or CBP capillary column (i. d. 0.5 mm x 25 m). TLC analyses were performed on commercial glass plates bearing 0.25-mm layer of Merck Silica gel $60F_{254}$. Silica gel (Wakosil C-200, Wako Pure Chemical Co.) was used for column chromatography. Gel permeation chromatography (GPC) was performed by LC-20AR (pump, SHIMADZU, 7.5 mL/min $CHCl_3$) and SPD-20A (UV detector, SHIMADZU, 254 nm) with two in-line YMC-GPC T2000 (20 x 600 mm, particle size: 10 μm) (preparative columns, YMC). UV-vis spectra

were acquired with JASCO V-750 spectrometer. Photoluminescence spectra and quantum yield measurements were conducted with JASCO FP-8500 spectrometer equipped with an integration sphere system. Cyclic voltammogram (CV) and differential pulse voltammetry (DPV) were recorded on ALS Electrochemical Analyzer Model 600E equipped with SVC-3 Voltammetry cell. Counter and working electrodes were made of Pt, and the reference electrode was Ag/Ag^+. The working electrodes were polished on a cloth polishing pad in an alumina slurry and then washed in H_2O under sonication before use. The measurements were conducted in *o*-dichlorobenzene/MeCN solvent (10/1, v/v) or dichloromethane (degassed by N_2 gas bubbling) containing tetrabutylammonium hexafluorophosphate as a supporting electrolyte at an indicated scan rate. All the potentials were calibrated with the standard ferrocene/ferrocenium (Fc/Fc^+) redox couple measured in identical conditions. The IUPAC convention was used to report the CV data.

Unless otherwise noted, materials obtained from commercial suppliers were used without further purification. All ammonium biarylborates **2** were prepared from the corresponding potassium trifluoroborates or pinacolboronates.[S1] All reactions were carried out under nitrogen atmosphere unless otherwise noted.

Caution!: Low molecular weight organoarsenic compounds are volatile and toxic. Although PhAs=O (1) is known as a bench-stable and non-hygroscopic solid, which ensures its suitability for safe handling in the laboratory,[S2] for safety, all experiments should be performed in a fume hood.

Experimental Procedures

Synthesis of **3a–3m** (**General Procedure A**): In a 20 mL screw cap test tube, phenylarsine oxide (**1**, 0.24 mmol, 40 mg) and biarylborate (**2**, 0.10 mmol) were placed with a magnetic stir bar. Toluene (1.5 mL) and 4-methylpyridine (0.36 mmol, 35 μL) were subsequently added by syringe. Finally, Tf_2O (0.24 mmol, 40 μl) was carefully added. The reaction mixture was heated at 110 ˚C in a heat block for 1 h. After cooling, sat. aq. $NaHCO_3$ was added. The resulting mixture was extracted three times with ethyl acetate (10 mL x 3). The combined organic layer was dried over Na_2SO_4 and concentrated in vacuo. The desired product dibenzoarsole **3** was isolated by column chromatography on silica gel using hexane/ethyl acetate as eluent followed by GPC with chloroform.

5-Phenyl-5*H*-benzo[*b*]arsindole (3a). Synthesized from **1** (0.24 mmol, 40 mg or 2.4 mmol, 400 mg), **2g** (0.10 mmol, 46 mg or 1.0 mmol, 460 mg), 4-methylpyridine (0.36 mmol, 35 μL or 3.6 mmol, 350 μL), Tf_2O (0.24 mmol, 40 μL or 2.4 mmol, 400 μL), and toluene (1.5 mL or 15 mL) according to **General Procedure A**. Purified by silica gel column chromatography with hexane/ethyl acetate (20/1, v/v) followed by GPC with chloroform:26 mg (85%, 0.10 mmol scale), 210 mg (69%, 1.0 mmol scale); white solid. The spectra data was in agreement with the reported values.[S3]

3-Methyl-5-phenyl-5*H*-benzo[*b*]arsindole (3b). Synthesized from **1** (0.24 mmol, 40 mg), **2b** (0.10 mmol, 48 mg), 4-methylpyridine (0.36 mmol, 35 μL), Tf_2O (0.24 mmol, 40 μL), and toluene (1.5 mL) according to **General Procedure A**. Purified by silica gel column chromatography with hexane/ethyl acetate (20/1, v/v) followed by GPC with chloroform: 18 mg (56%, 0.10 mmol scale); white solid; m.p. 90.3-91.3 °C; ^{1}H NMR ($CDCl_3$, 400 MHz): δ 7.88 (d, J = 7.7 Hz, 1H), 7.80 (d, J = 7.9 Hz, 1H), 7.68-7.70 (m, 1H), 7.520-7.524 (m, 1H), 7.43 (ddd, J = 7.7, 7.7, 1.2 Hz, 1H), 7.23-7.29 (m, 4H), 7.16-7.23 (m, 3H), 2.38 (s, 3H). $^{13}C\{^1H\}$ NMR ($CDCl_3$, 100 MHz): δ 146.0 (1C), 145.8 (1C), 145.5 (1C), 143.0 (1C), 140.2 (1C), 137.8 (1C), 132.4 (2C), 132.2 (1C), 131.7 (1C), 129.8 (1C), 128.81 (2C), 128.77 (1C), 128.6 (1C), 127.5 (1C), 122.0 (1C), 121.95(1C), 21.6 (1C). HRMS (APCI) m/z $(M+H)^+$ calcd for $C_{19}H_{16}As$: 319.0462, found: 319.0472.

3-Methoxy-5-phenyl-5*H*-benzo[*b*]arsindole (3c). Synthesized from **1** (0.24 mmol, 40 mg), **2c** (0.10 mmol, 52 mg), 4-methylpyridine (0.36 mmol, 35 μL), Tf_2O (0.24 mmol, 40 μL), and toluene (1.5 mL) according to **General Procedure A**. Purified by silica gel column chromatography with hexane/ethyl

acetate (20/1, v/v) followed by GPC with chloroform: 24 mg (72%, 0.10 mmol scale); white solid; m.p. 106.1-107.1 °C; ^{1}H NMR ($CDCl_3$, 400 MHz): δ 7.82 (d, J = 8.4 Hz, 1H), 7.80 (d, J = 6.3 Hz, 1H), 7.67 (dd, J = 7.3 0.5 Hz, 1H), 7.42 (ddd, J = 7.5, 7.5, 1.2 Hz, 1H), 7.28-7.16 (m, 7H), 6.98 (dd, J = 8.5, 2.5 Hz, 1H), 3.83 (s, 3H). ^{13}C{^{1}H} NMR ($CDCl_3$, 100 MHz): δ 159.6 (1C), 147.6 (1C), 145.6 (1C), 145.0 (1C), 140.2 (1C), 138.5 (1C), 132.4 (2C), 131.6 (1C), 128.85 (2C), 128.81 (1C), 128.7 (1C), 126.8 (1C), 123.1 (1C), 121.5 (1C), 116.3 (1C), 115.2 (1C), 55.6 (1C). HRMS (APCI) m/z $(M+H)^+$ calcd for $C_{19}H_{16}AsO$: 335.0412, found: 335.0399.

3-(*tert*-Butyl)-5-phenyl-5*H*-benzo[*b*]arsindole (3d). Synthesized from **1** (0.24 mmol, 40 mg), **2d** (0.10 mmol, 54 mg), 4-methylpyridine (0.36 mmol, 35 μL), Tf_2O (0.24 mmol, 40 μL), and toluene (1.5 mL) according to **General Procedure A**. Purified by silica gel column chromatography with hexane/ethyl acetate (20/1, v/v) followed by GPC with chloroform: 20 mg (56%, 0.10 mmol scale); colorless oil; ^{1}H NMR ($CDCl_3$, 400 MHz): δ 7.87 (d, J = 7.7 Hz, 1H), 7.84 (d, J = 8.2 Hz, 1H), 7.75 (d, J = 1.8 Hz, 1H), 7.69 (dd, J = 7.5, 0.56 Hz, 1H), 7.48 (dd, J = 8.2, 2.0 Hz, 1H), 7.42 (ddd, J = 7.5, 7.5, 1.2 Hz, 1H), 7.29-7.24 (m, 3H), 7.21-7.16 (m, 3H), 1.34 (s, 9H). ^{13}C{^{1}H} NMR ($CDCl_3$, 100 MHz): δ 151.2 (1C), 145.8 (1C), 145.6 (1C), 145.5 (1C), 143.1 (1C), 140.4 (1C), 132.4 (2C), 131.6 (1C), 128.8 (2C), 128.7 (1C), 128.5 (1C), 128.4 (1C), 127.5 (1C), 126.2 (1C), 122.0 (1C), 121.8 (1C), 35.1 (1C), 31.6 (3C). HRMS (APCI) m/z $(M+H)^+$ calcd for $C_{22}H_{22}As$: 361.0932, found: 361.0935.

3,5-Diphenyl-5*H*-benzo[*b*]arsindole (3e). Synthesized from **1** (0.24 mmol, 40 mg), **2e** (0.10 mmol, 49 mg), 4-methylpyridine (0.36 mmol, 35 μL), Tf_2O (0.24 mmol, 40 μL), and toluene (1.5 mL) according to **General Procedure A**. Purified by silica gel column chromatography with hexane/ethyl acetate (20/1, v/v) followed by GPC with chloroform: 24 mg (62%, 0.10 mmol scale); white solid; m.p. 134.0–135.0 °C; ^{1}H NMR ($CDCl_3$, 400 MHz): δ 8.00-7.95 (m, 3H), 7.76-7.73 (m, 1H), 7.70 (dd, J = 8.1, 1.8 Hz, 1H), 7.65-7.63 (m, 2H), 7.50-7.43 (m, 3H), 7.38-7.30 (m, 4H), 7.23-7.18 (m, 3H). ^{13}C{^{1}H} NMR ($CDCl_3$, 100 MHz): δ 146.6 (1C), 146.2 (1C), 145.4 (1C), 144.7 (1C), 140.9 (1C), 140.8 (1C), 140.0 (1C), 132.4 (2C), 131.7 (1C), 130.2 (1C), 128.95 (2C), 128.90 (1C), 128.8 (2C), 128.7 (1C), 127.9 (1C), 127.8 (1C), 127.5 (1C), 127.2 (2C), 122.5 (1C), 122.4 (1C). HRMS (APCI) m/z $(M+H)^+$ calcd for $C_{24}H_{18}As$: 381.0619, found: 381.0617.

3-Chloro-5-phenyl-5*H*-benzo[*b*]arsindole (3f). Synthesized from **1** (0.24 mmol, 40 mg), **2f** (0.10 mmol, 50 mg), 4-methylpyridine (0.36 mmol, 35 μL), Tf_2O (0.24 mmol, 40 μL), and toluene (1.5 mL) according

to **General Procedure A**. Purified by silica gel column chromatography with hexane/ethyl acetate (20/1, v/v) followed by GPC with chloroform: 16 mg (47%, 0.10 mmol scale); white solid; m.p. 78.0–79.0 ºC; ^{1}H NMR ($CDCl_3$, 400 MHz): *δ* 7.88 (d, *J* = 7.8 Hz, 1H), 7.83 (d, *J* = 8.3 Hz, 1H), 7.71 (dd, *J* = 7.4. 0.52 Hz, 1H), 7.66 (d, *J* = 2.0 Hz, 1H), 7.47 (ddd, *J* = 7.5, 7.5, 1.2 Hz, 1H), 7.41 (dd, *J* = 8.3, 2.1 Hz, 1H), 7.33 (ddd, *J* = 7.4, 7.4, 1.2 Hz, 1H), 7.25-7.20 (m, 5H). ^{13}C{^{1}H} NMR ($CDCl_3$, 100 MHz): *δ* 148.0 (1C), 145.6 (1C), 144.7 (1C), 144.0 (1C), 139.3 (1C), 133.7 (1C), 132.4 (2C), 131.8 (1C), 131.4 (1C), 129.1 (1C), 129.03 (1C), 128.97 (3C, overlapped), 128.2 (1C), 123.2 (1C), 122.4 (1C). HRMS (APCI) m/z $(M+H)^+$ calcd for $C_{18}H_{13}AsCl$: 338.9916, found: 338.9921.

5-Phenyl-3-(trifluoromethyl)-5*H*-benzo[*b*]arsindole (3g). Synthesized from **1** (0.24 mmol, 40 mg), **2g** (0.10 mmol, 53 mg), 4-methylpyridine (0.36 mmol, 35 μL), Tf_2O (0.24 mmol, 40 μL), and toluene (1.5 mL) according to **General Procedure A**. Purified by silica gel column chromatography with hexane/ethyl acetate (20/1, v/v) followed by GPC with chloroform: 20 mg (54%, 0.10 mmol scale); white solid; m.p. 47.1-48.1 ºC; ^{1}H NMR ($CDCl_3$, 400 MHz): *d* 8.02-7.95 (m, 3H), 7.76-7.69 (m, 2H), 7.47 (ddd, *J* = 7.7, 7.7, 1.2 Hz, 1H), 7.47 (ddd, *J* = 7.4, 7.4, 1.1 Hz, 1H) , 7.33 (d, *J* = 3.3 Hz, 1H), 7.27-7.19 (m, 4H). ^{13}C{^{1}H} NMR ($CDCl_3$, 100 MHz): *δ* 149.0 (1C), 146.8 (1C), 146.7 (1C), 144.3 (1C), 138.9 (1C), 132.4 (2C), 131.9 (1C), 129.6 (q, *J* = 32.1 Hz, 1C), 129.1 (1C), 129.0 (4C, overlapped), 128.4 (q, *J* = 4.1 Hz, 1C), 126.0 (q, *J* = 3.7 Hz, 1C), 124.4 (q, *J* = 270.3 Hz, 1C), 123.1 (1C), 122.3 (1C). ^{19}F{^{1}H} NMR ($CDCl_3$, 376 MHz): *δ* -62.0. HRMS (APCI) m/z $(M+H)^+$ calcd for $C_{19}H_{13}AsF_3$: 373.0180, found: 373.0181.

7-Methoxy-2-methyl-5-phenyl-5*H*-benzo[*b*]arsindole (3h). Synthesized from **1** (0.24 mmol, 40 mg), **2h** (0.10 mmol, 51 mg), triethylamine (0.36 mmol, 50 μL) instead of 4-methylpyridine, Tf_2O (0.24 mmol, 40 μL), and toluene (1.5 mL) according to **General Procedure A but slightly modified with temperature: the reaction temperature was kept at 0 ˚C when Tf_2O was added, and the resulting solution was stirred at 60 ˚C**. Purified by silica gel column chromatography with hexane/ethyl acetate (20/1, v/v) followed by GPC with chloroform; white solid: 18 mg (52%, 0.10 mmol scale); white solid; m.p. 126.2-127.9 ˚C; ^{1}H NMR (400 MHz, $CDCl_3$) δ 7.81 (d, *J* = 8.6 Hz, 1H), 7.63 (s, 1H), 7.55 (d, *J* = 7.6 Hz, 1H), 7.29-7.26 (m, 2H), 7.24 (d, *J* = 2.4 Hz, 1H), 7.22-7.16 (m, 3H), 7.07 (dq, *J* = 7.6, 0.7 Hz, 1H), 6.98 (dd, *J* = 8.5, 2.5 Hz, 1H), 3.83 (s, 3H), 2.44 (s, 3H); ^{13}C{^{1}H} NMR (100 MHz, $CDCl_3$) δ 159.4 (1C), 147.9 (1C), 145.7 (1C), 141.4 (1C), 140.3 (1C), 138.6 (1C), 138.3 (1C), 132.2 (2C), 131.3 (1C), 128.7 (2C), 128.5 (1C), 127.8 (1C), 122.9 (1C), 122.2 (1C), 116.2 (1C), 114.9 (1C), 55.5 (1C), 21.7 (1C);

HRMS (APCI) m/z ($[M+H]^+$) calcd for $C_{20}H_{18}AsO$: 349.0568, found: 349.0591.

11-Phenyl-11*H*-dibenzo[*b,g*]arsindole (3i). Synthesized from **1** (0.24 mmol, 40 mg), **2i** (0.10 mmol, 51 mg), 4-methylpyridine (0.36 mmol, 35 μL), Tf_2O (0.24 mmol, 40 μL), and toluene (1.5 mL) according to **General Procedure A**. Purified by silica gel column chromatography with hexane/ethyl acetate (20/1, v/v) followed by GPC with chloroform; white solid: 26 mg (72%, 0.10 mmol scale); m.p. 137.0-138.0 °C; ^{1}H NMR ($CDCl_3$, 400 MHz): *δ* 8.05 (d, *J* = 8.5, 1H), 8.00-7.94 (m, 3H), 7.89-7.86 (m, 1H), 7.76-7.74 (m, 1H), 7.50-7.43 (m, 3H), 7.34-7.28 (m, 3H), 7.20-7.12 (m, 3H). $^{13}C\{^1H\}$ NMR ($CDCl_3$, 100 MHz): *δ* 146.4 (1C), 146.3 (1C), 144.8 (1C), 144.0 (1C), 139.0 (1C), 135.0 (1C), 133.2 (1C), 132.8 (2C), 131.5 (1C), 130.0 (1C), 128.9 (3C, overlapped), 128.8 (2C), 128.1 (1C), 127.9 (1C), 127.2 (1C), 126.2 (1C), 122.5 (1C), 120.6 (1C). HRMS (APCI) m/z $(M+H)^+$ calcd for $C_{22}H_{16}As$: 355.0462, found: 355.0465.

10-Phenyl-10*H*-arsindolo[3,2-*b*]benzo[*d*]thiophene (3j).[S4] Synthesized from **1** (0.24 mmol, 40 mg), **2j** (0.10 mmol, 52 mg), 4-methylpyridine (0.36 mmol, 35 μL), Tf_2O (0.24 mmol, 40 μL), and toluene (1.5 mL) according to **General Procedure A**. Purified by silica gel column chromatography with hexane/ethyl acetate (20/1, v/v) followed by GPC with chloroform: 26 mg (71%, 0.10 mmol scale); white solid; m.p. 116.3-117.3 °C; ^{1}H NMR ($CDCl_3$, 400 MHz): *δ* 7.91-7.89 (m, 1H), 7.77-7.74 (m, 1H), 7.71-7.67 (m, 2H), 7.42 (ddd, *J* = 7,5, 7.5, 1.2 Hz, 1H), 7.37-7.27 (m, 5H), 7.24-7.18 (m, 3H). $^{13}C\{^1H\}$ NMR ($CDCl_3$, 100 MHz): *δ* 150.1 (1C), 148.4 (1C), 142.5 (1C), 141.6 (1C), 140.1 (1C), 139.7 (1C), 138.4 (1C), 132.5 (2C), 131.4 (1C), 129.02 (3C, overlapped), 128.96 (1C), 127.3 (1C), 125.2 (1C), 124.6 (1C), 123.54 (1C), 123.52 (1C), 122.6 (1C). HRMS (APCI) m/z $(M+H)^+$ calcd for $C_{20}H_{14}AsS$: 361.0027, found: 361.0015.

6-Phenyl-6*H*-arsindolo[2,3-*b*]benzo[*d*]thiophene (3k). Synthesized from **1** (0.24 mmol, 40 mg), **2k** (48 0.10 mmol, 52 mg), 4-methylpyridine (0.36 mmol, 35 μL), Tf_2O (0.24 mmol, 40 μL), and toluene (1.5 mL) according to **General Procedure A**. Purified by silica gel column chromatography with hexane/ethyl acetate (20/1, v/v) followed by GPC with chloroform: 26 mg (71%, 0.10 mmol scale); white solid; m.p. 109.0-110.0 °C; ^{1}H NMR ($CDCl_3$, 400 MHz): *δ* 8.45 (d, *J* = 8.2 Hz, 1H), 8.23 (d, *J* = 7.7 Hz, 1H), 7.91 (d, *J* = 8.1 Hz, 1H), 7.67 (dd, *J* = 7.4, 0.68 Hz, 1H), 7.53-7.47 (m, 2H), 7.41-7.37 (m, 1H), 7.34-7.31 (m, 2H), 7.27-7.21 (m, 4H). $^{13}C\{^1H\}$ NMR ($CDCl_3$, 100 MHz): *δ* 150.2 (1C), 147.5 (1C), 147.0

(1C), 146.0 (1C), 142.7 (1C), 138.7 (1C), 135.3 (1C), 132.5 (2C), 131.5 (1C), 129.2 (1C), 129.0 (2C), 128.8 (1C), 126.1 (1C), 124.8 (1C), 124.4 (1C), 123.5 (1C), 122.4 (1C), 122.3 (1C). HRMS (APCI) m/z $(M+H)^+$ calcd for $C_{20}H_{14}AsS$: 361.0027, found: 361.0020.

10-Phenyl-10*H*-phenoxarsinine (3l).[S5] Synthesized from **1** (0.24 mmol, 40 mg), **2l** (0.10 mmol, 48 mg), 4-methylpyridine (0.36 mmol, 35 μL), Tf_2O (0.24 mmol, 40 μL), and toluene (1.5 mL) according to **General Procedure A**. Purified by silica gel column chromatography with hexane/ethyl acetate (20/1, v/v) followed by GPC with chloroform: 25 mg (77%, 0.10 mmol scale); white solid; m.p. 108.5-109.5 °C; ^{1}H NMR ($CDCl_3$, 400 MHz): δ 7.53 (dd, J = 7.4, 1.6 Hz, 2H), 7.39-7.35 (m, 2H), 7.26-7.23 (m, 4H), 7.21-7.19 (m, 3H), 7.12 (ddd, J = 7.3, 7.3, 1.2 Hz, 2H). $^{13}C\{^1H\}$ NMR ($CDCl_3$, 100 MHz): δ 154.9 (2C), 142.1 (1C), 135.0 (2C), 132.2 (2C), 130.9 (2C), 128.7 (2C), 128.4 (1C), 123.9 (2C), 120.8 (2C), 118.5 (2C). HRMS (APCI) m/z $(M+H)^+$ calcd for $C_{18}H_{14}AsO$: 321.0255, found: 321.0251.

5,10-Diphenyl-5,10-dihydrophenarsazinine (3m).[S6] Synthesized from **1** (0.24 mmol, 40 mg), **2m** (0.10 mmol, 55 mg), 4-methylpyridine (0.36 mmol, 35 μL), Tf_2O (0.24 mmol, 40 μL), and toluene (1.5 mL) according to **General Procedure A**. Purified by silica gel column chromatography with hexane/ethyl acetate (20/1, v/v) followed by GPC with chloroform: 21 mg (59%, 0.10 mmol scale); white solid; m.p. 128.5-129.5 °C; ^{1}H NMR ($CDCl_3$, 400 MHz): δ 7.67 (dd, J = 7.2, 1.7 Hz, 2H), 7.53-7.49 (m, 2H), 7.45-7.41 (m, 1H), 7.18-7.06 (m, 7H), 7.02-6.96 (m, 4H), 6.51 (dd, J = 8.4, 0.88 Hz, 2H). $^{13}C\{^1H\}$ NMR ($CDCl_3$, 100 MHz): δ 145.3 (2C), 143.6 (1C), 142.7 (1C), 135.6 (2C), 131.5 (2C), 131.2 (2C), 130.7 (2C), 129.8 (2C), 128.4 (2C), 128.1 (1C), 127.7 (1C), 121.4 (2C), 121.2 (2C), 117.6 (2C). HRMS (APCI) m/z $(M+H)^+$ calcd for $C_{24}H_{19}AsN$: 396.0728, found: 396.0717.

Synthesis of **3n**: In a 20 mL Schlenk tube, phenylarsine oxide (**1**, 0.24 mmol, 40 mg) and biarylborate (**2n**, 0.05 mmol, 35 mg) were placed with a magnetic stir bar. Toluene (1.5 mL) and 4-methylpyridine (0.36 mmol, 35 μL) were subsequently added by syringe. Finally, Tf_2O (0.24 mmol, 40 μL) was carefully added. The reaction mixture was heated at 110 °C in a heat block for 2 h. After cooling, sat. aq. $NaHCO_3$ was added. The resulting mixture was extracted three times with chloroform (10 mL x 3). The combined organic layer was filtered through a short pad of silica gel and concentrated in vacuo. The desired product 8,16-diphenylbenzo[*b*]thieno[2,3-*d*]arsindolo[1,2-*a*]benzo[*b'*]thieno[2',3'-*a'*]arsindolo[1',2'-*e*]naphthalene (**3n**) was isolated in an analytically pure form by washing with cooled

chloroform.

***trans*-8,16-Diphenylbenzo[*b*]thieno[2,3-*d*]arsindolo[1,2-*a*]benzo[*b'*]thieno[2',3'-*a'*]arsindolo[1',2'-*e*]naphthalene (3n).** Purified by washing with cooled chloroform: 21 mg (59%, 0.05 mmol scale); yellow solid; m.p. > 300 °C; ^{1}H NMR (CD_2Cl_2, 400 MHz): δ 8.44 (d, J = 8.2 Hz, 2H), 8.36 (d, J = 8.5 Hz, 2H), 8.02 (d, J = 8.5 Hz, 2H), 7.88 (d, J = 8.0 Hz, 2H), 7.49-7.45 (m, 2H), 7.36-7.32 (m, 6H), 7.18-7.12 (m, 6H). ^{13}C{^{1}H} NMR (CD_2Cl_2, 100 MHz): The clear spectra was not obtained because of the solubility issue of the compound. HRMS (APCI) m/z (M)$^+$ calcd for $C_{38}H_{22}As_2S_2$: 691.9589, found: 691.957. The suitable crystals for X-ray analysis were grown from $CHCl_3$/hexane.

Oxidation of **3a** with H_2O_2: A Schlenk tube equipped with a magnetic stir bar was charged with 5-phenyl-5*H*-benzo[*b*]arsindole (**3a**, 0.65 mmol, 198 mg) and THF (2.0 mL). aq. H_2O_2 (30%) was added dropwise, with a total of 15 drops being introduced. After being stirred for 48 hours at room temperature, the mixture was quenched with aq. $Na_2S_2O_3$. The resulting mixture was extracted three times with chloroform (10 mL x 3). The combined organic layer was dried over Na_2SO_4 and concentrated in vacuo. The desired product 5-phenylbenzo[*b*]arsindole 5-oxide (**4a**; 190 mg, 0.59 mmol, 91%) was isolated by column chromatography on silica gel using ethyl acetate/methanol (10/1, v/v) as eluent followed by GPC with chloroform.

5-Phenylbenzo[*b*]arsindole 5-oxide (4a).[S7] Purified by silica gel column chromatography with ethyl acetate/methanol (10/1, v/v) followed by GPC with chloroform: 190 mg (91%, 0.65 mmol scale); white solid; m.p. 180.1-181.1 °C; ^{1}H NMR ($CDCl_3$, 400 MHz): δ 7.95 (d, J = 7.8 Hz, 2H), 7.79 (d, J = 7.4, 2H), 7.69-7.62 (m, 4H), 7.55-7.51 (m, 1H), 7.48-7.44 (m, 4H). ^{13}C{^{1}H} NMR ($CDCl_3$, 100 MHz): δ 141.1 (2C), 133.3 (2C), 132.6 (2C), 132.5 (1C), 132.4 (1C), 130.69 (2C), 130.67 (2C), 130.2 (2C), 129.6 (2C), 122.4 (2C). HRMS (APCI) m/z (M+H)$^+$ calcd for $C_{18}H_{14}AsO$: 321.0255, found: 321.0274.

Arsa-Baeyer-Villiger oxidation of **4a** with *m*CPBA: A Schlenk tube equipped with a magnetic stir bar was charged with 5-phenylbenzo[*b*]arsindole 5-oxide (**4a**, 0.05 mmol, 16 mg) and THF (1.0 mL). *m*-Chloroperoxybenzoic acid (*m*CPBA; *ca.* 70%, 0.15 mmol, 26 mg) was added to the mixture. After being stirred overnight at room temperature, the mixture was quenched with aq. $Na_2S_2O_3$. The resulting

mixture was extracted three times with chloroform (10 mL x 3). The combined organic layer was dried over Na_2SO_4 and concentrated in vacuo. The desired product 6-phenyldibenzo[*c*,*e*][1,2]oxarsinine 6-oxide (**5a**; 14 mg, 0.043 mmol, 86%) was isolated in an analytically pure form by washing with ethyl acetate.

6-Phenyldibenzo[*c*,*e*][1,2]oxarsinine 6-oxide (5a). Purified by washing with ethyl acetate: 14 mg (86%, 0.05 mmol scale); white solid; m.p. 280.1-281.1 °C; ^{1}H NMR ($CDCl_3$, 400 MHz): δ 8.09 (d, J = 7.7 Hz, 1H), 7.89-7.87 (m, 2H), 7.75 (d, J = 7.6 Hz, 1H), 7.60 (dd, J = 7.9, 1.4 Hz, 1H), 7.57-7.53 (m, 1H), 7.41-7.37 (m, 1H), 7.30 (dd, J = 7.4, 7.4 Hz, 2H), 7.26-7.24 (m, 1H), 7.24-7.19 (m, 1H), 7.00 (dd, J = 8.2, 1.1 Hz, 1H), 6.98-6.94 (m, 1H). ^{13}C{^{1}H} NMR ($CDCl_3$, 100 MHz): δ 156.4 (1C), 140.0 (1C), 136.5 (1C), 132.9 (1C), 132.7 (1C), 132.1 (2C), 131.8 (1C), 131.7 (1C), 130.4 (1C), 128.8 (2C), 127.4 (1C), 126.1 (2C, overlapped), 122.9 (1C), 121.7 (1C), 121.1 (1C). HRMS (APCI) m/z $(M+H)^+$ calcd for $C_{18}H_{14}AsO_2$: 337.0204, found: 337.0204.

Sequential oxidation of **3b** with H_2O_2 and *m*CPBA: A Schlenk tube equipped with a magnetic stir bar was charged with 3-methyl-5-phenyl-5*H*-benzo[*b*]arsindole (**3b**, 0.042 mmol, 13.5 mg) and THF (1.0 mL). aq. H_2O_2 (30%) was added dropwise, with a total of 5 drops being introduced. After being stirred for 12 hours at room temperature, the reaction was quenched with aq. $Na_2S_2O_3$. The resulting mixture was extracted three times with chloroform (10 mL x 3). The combined organic layer was dried over Na_2SO_4 and concentrated in vacuo. The residue was placed with another Schlenk tube equipped with a magnetic stir bar, which was dissolved in THF (1 mL). *m*-Chloroperoxybenzoic acid (*m*CPBA, *ca.* 70%, 0.15 mmol, 26 mg) was then added. After being stirred for 12 hours at room temperature, the mixture was quenched with aq. $Na_2S_2O_3$. The resulting mixture was extracted three times with chloroform (10 mL x 3). The combined organic layer was dried over Na_2SO_4 and concentrated in vacuo. The desired products, 8-methyl-6-phenyldibenzo[*c,e*][1,2]oxarsinine 6-oxide and 3-methyl-6-phenyldibenzo[*c,e*][1,2]oxarsinine 6-oxide, (**5b**, 14 mg, 0.043 mmol, 94%, 54:46) were isolated in analytically pure forms by washing with hexane.

8-Methyl-6-phenyldibenzo[*c,e*][1,2]oxarsinine 6-oxide and 3-methyl-6-phenyldibenzo[*c,e*][1,2]oxarsinine 6-oxide (5b) Purified by washing with hexane: 14 mg (94%, 0.04 mmol scale); white solid; m.p. 119.5-121.1 °C; ^{1}H NMR ($CDCl_3$, 400 MHz): δ 8.01 (d, J = 7.4 Hz, 0.54H), 7.95-7.92 (m,

2H), 7.81-7.79 (m, 1H), 7.69 (d, *J* = 8.2 Hz, 0.54H), 7.64-7.57 (m, 1.38H), 7.49-7.46 (m, 1H), 7.40-7.36 (m, 2.54H), 7.30-7.27 (m, 0.46H), 7.24- 7.20 (m, 0.54H), 7.05-6.97 (m, 1H), 6.88-6.84 (m, 1H), 2.33 (s, 1.62 H), 2.28 (s, 1.38 H); $^{13}C\{^1H\}$ NMR ($CDCl_3$, 100 MHz): δ 140.8, 139.7, 137.5, 136.8, 133.6, 132.6, 132.5, 132.02, 131.98, 131.9, 129.9, 128.8, 128.7, 127.1, 125.8, 125.5, 122.8, 122.5, 121.8, 121.5, 121.2, 119.8, 21.3, 21.2 (Several signals were overlapped, and all signals could not be completely assigned. Thus, all observed signals were just shown.); HRMS (APCI) m/z ($[M+H]^+$) calcd for $C_{19}H_{16}AsO_2$: 351.0361 found: 351.0360.

Synthesis of **6a** and **6b**:[S8] In a glovebox filled with nitrogen, $Ni(cod)_2$ (2.8 mg, 0.010 mmol, 0.10 equiv), IPr･HCl (12.8 mg, 0.030 mmol, 0.30 equiv), NaO*t*Bu (0.036 mmol, 0.36 equiv), and toluene (0.20 mL) were added to a Schlenk tube with pressure resistance. The tube was taken out from the glovebox, and the solution was stirred for 5 min at rt. 5-Phenylbenzo[*b*]arsindole 5-oxide **4a** (0.10 mmol, 1.0 equiv) and $AlMe_3$ (1.4 M in hexane, 0.14 mL, 2.0 equiv) or $AlEt_3$ (1.0 M in hexane, 0.20 mL, 2.0 equiv) were then added. The tube was heated at 180 °C for 18 h (oil bath). After the reaction mixture was cooled to rt, the crude mixture was filtered through a pad of Celite and Na_2SO_4 eluting with EtOAc and evaporation under reduced pressure formed a crude material. The residue was purified by column chromatography on silica gel with hexane and GPC ($CHCl_3$) to give the corresponding 5-alkyldibenzoarsole derivative **6**.

5-Methyl-5*H*-benzo[*b*]arsindole (6a).[S3] 9.5 mg (39%, 0.10 mmol scale); colorless oil; ^1H NMR (400 MHz, $CDCl_3$) δ 7.91 (d, *J* = 7.8 Hz, 2H), 7.72 (dq, *J* = 7.4, 0.56 Hz, 2H), 7.44 (td, *J* = 7.4, 1.2 Hz, 2H), 7.31 (td, *J* = 7.4, 1.2 Hz, 2H), 1.27 (s, 3H); $^{13}C\{^1H\}$ NMR (100 MHz, $CDCl_3$) δ 147.8 (2C), 145.1 (2C), 130.8 (2C), 128.3 (2C), 127.3 (2C), 122.1 (2C), 12.8 (1C); HRMS (APCI) m/z ($[M+H]^+$) calcd for $C_{13}H_{12}As$: 243.0149, found: 243.0140.

5-Ethyl-5*H*-benzo[*b*]arsindole (6b): 9.8 mg (38%, 0.10 mmol scale); colorless oil; ^1H NMR (400 MHz, $CDCl_3$) δ 7.90 (d, *J* = 7.8 Hz, 2H), 7.70 (dq, *J* = 7.4, 0.56 Hz, 2H), 7.44 (td, *J* = 7.5, 1.3 Hz, 2H), 7.31 (td, *J* = 7.3, 1.1 Hz, 2H), 1.71 (q, *J* = 7.7 Hz, 2H), 0.98 (t, *J* = 7.6 Hz, 3H); $^{13}C\{^1H\}$ NMR (100 MHz, $CDCl_3$) δ 145.9 (2C), 145.7 (2C), 131.1 (2C), 128.2 (2C), 127.2 (2C), 122.0 (2C), 22.8 (1C), 10.5 (1C); HRMS (APCI) m/z ($[M+H]^+$) calcd for $C_{14}H_{14}As$: 257.0306, found: 257.0316.

Detailed Optimization Studies

Table S1. Optimization studies for direct synthesis of dibenzoarsole **3a** from phenylarsine oxide **1** and ammonium biphenylborate **2a**

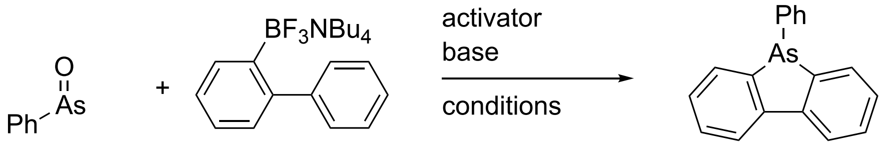

1, x mmol **2a**, y mmol **3a**

entry	x (mmol)	y (mmol)	activator (mmol)	base (mmol)	solvent (mL)	temp (°C)	time	NMR yield[a]
1	0.20	0.10	Tf_2O (0.20)	NEt_3 (0.30)	TCE (1.5)	90	1 h	58%
2	***0.20***	***0.10***	***Tf_2O (0.20)***	***NEt_3 (0.30)***	***toluene (1.5)***	***90***	***1 h***	***59%***
3	0.20	0.10	Tf_2O (0.20)	NEt_3 (0.30)	cyclohexane (1.5)	90	1 h	32%
4	0.20	0.10	Tf_2O (0.20)	NEt_3 (0.30)	toluene (1.5)	80	1 h	58%
5	0.20	0.10	Tf_2O (0.20)	NEt_3 (0.30)	toluene (1.5)	70	1 h	trace
6	0.20	0.10	Tf_2O (0.20)	NEt_3 (0.30)	toluene (1.5)	100	1 h	74%
7	***0.20***	***0.10***	***Tf_2O (0.20)***	***NEt_3 (0.30)***	***toluene (1.5)***	***110***	***1 h***	***78%***
8	0.20	0.10	Tf_2O (0.20)	NEt_3 (0.30)	toluene (1.5)	120	1 h	75%
9	0.20	0.10	Tf_2O (0.20)	NEt_3 (0.30)	toluene (1.5)	110	0.5 h	76%
10	0.20	0.10	Tf_2O (0.20)	NEt_3 (0.30)	toluene (1.5)	110	1.5 h	63%
11	0.18	0.10	Tf_2O (0.18)	NEt_3 (0.27)	toluene (1.5)	110	1 h	74%
12	***0.24***	***0.10***	***Tf_2O (0.24)***	***NEt_3 (0.36)***	***toluene (1.5)***	***110***	***1 h***	***86% (80%)***
13	0.30	0.10	Tf_2O (0.3)	NEt_3 (0.45)	toluene (1.5)	110	1 h	78%
14	0.24	0.10	Tf_2O (0.24)	pyridine (0.36)	toluene (1.5)	110	1 h	75%
15	0.24	0.10	Tf_2O (0.24)	DMAP (0.36)	toluene (1.5)	110	1 h	33%
16	0.24	0.10	Tf_2O (0.24)	2,6-lutidine (0.36)	toluene (1.5)	110	1 h	76%
17	***0.24***	***0.10***	***Tf_2O (0.24)***	***4-methylpyridine (0.36)***	***toluene (1.5)***	***110***	***1 h***	***87% (85%)***
18	0.24	0.10	Tf_2O (0.24)	DBU (0.36)	toluene (1.5)	110	1 h	n.d.
19	0.24	0.10	Tf_2O (0.24)	DABCO (0.36)	toluene (1.5)	110	1 h	n.d.
20	0.24	0.10	Tf_2O (0.24)	K_2CO_3 (0.36)	toluene (1.5)	110	1 h	71%
21	0.24	0.10	Tf_2O (0.24)	Cs_2CO_3 (0.36)	toluene (1.5)	110	1 h	37%
22	0.24	0.10	Tf_2O (0.24)	K_3PO_4 (0.36)	toluene (1.5)	110	1 h	45%
23	0.24	0.10	Tf_2O (0.24)	KOH (0.36)	toluene (1.5)	110	1 h	61%
24	0.24	0.10	Tf_2O (0.24)	none	toluene (1.5)	110	1 h	44%
25	0.24	0.10	Ts_2O (0.24)	4-methylpyridine (0.36)	toluene (1.5)	110	1 h	n.d.
26	0.24	0.10	TfOH (0.48)	none	toluene (1.5)	110	1 h	trace
27	0.24	0.10	$PhNTf_2$ (0.24)	4-methylpyridine (0.36)	toluene (1.5)	110	1 h	n.d.
28	0.24	0.10	$(CH_3CO)_2O$ (0.24)	4-methylpyridine (0.36)	toluene (1.5)	110	1 h	n.d.
29	0.24	0.10	$(CF_3CO)_2O$ (0.24)	4-methylpyridine (0.36)	toluene (1.5)	110	1 h	n.d.
30	0.24	0.10	Tf_2O (0.24)	4-methylpyridine (0.36)	TCE (1.5)	110	1 h	68%
31	0.24	0.10	Tf_2O (0.24)	4-methylpyridine (0.36)	$PhCF_3$ (1.5)	110	1 h	n.d.
32	0.24	0.10	Tf_2O (0.24)	4-methylpyridine (0.36)	DMF (1.5)	110	1 h	65%
33	0.24	0.10	Tf_2O (0.24)	4-methylpyridine (0.36)	1,4-dioxane (1.5)	110	1 h	82%

[a]NMR yield using triethylphosphte as an internal standard. Isolated yields are in parentheses.

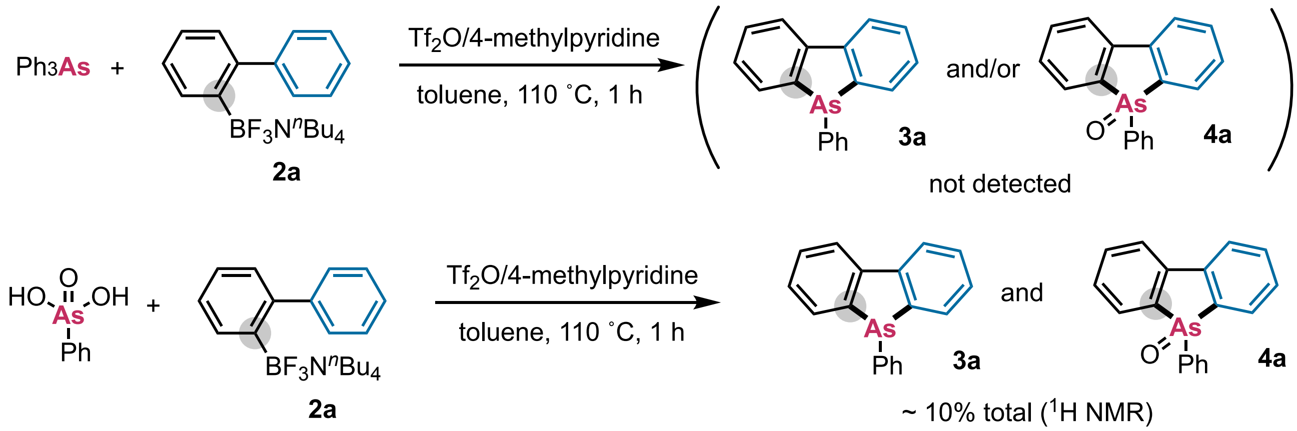

Scheme S1. Attempts to apply arsenic sources other than PhAs=O (**1**).

X-Ray Analysis

The single X-ray quality crystals of **3n** were grown from $CHCl_3$/hexane by slow evaporation at room temperature. The structure was refined by full-matrix least-squares method using SHELXL-2017/1.

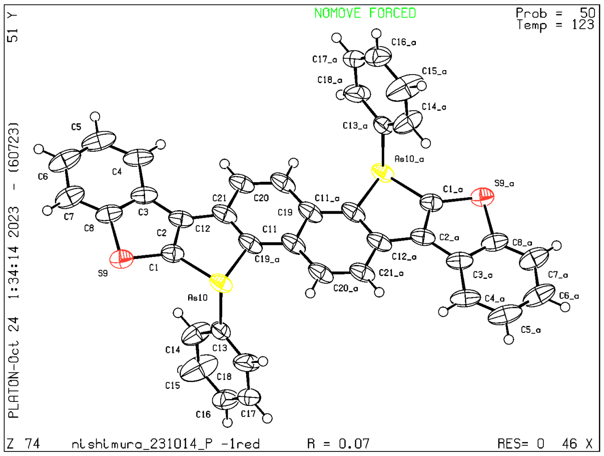

Figure S1. ORTEP drawing of **3n** (CCDC 2329867, 50% thermal probability).

Table S2. Crystal Data for **3n**

Crystal system	triclinic
Space group IT number	2
Space group name H-M alt	P -1
Space group name Hall	-P 1
Cell length a	7.6593(5)
Cell length b	8.7731(6)
Cell length c	11.3505(8)
Cell angle alpha	70.661(6)
Cell angle beta	83.531(6)
Cell angle gamma	83.032(6)
Cell volume	712.21(9)
Cell formula units Z	2
Refine ls R factor all	0.0753
Refine ls R factor gt	0.0697
Refine ls wR factor gt	0.1810
Refine ls wR factor ref	0.1901
Refine ls goodness of fit ref	1.049

Photoluminescence Properties

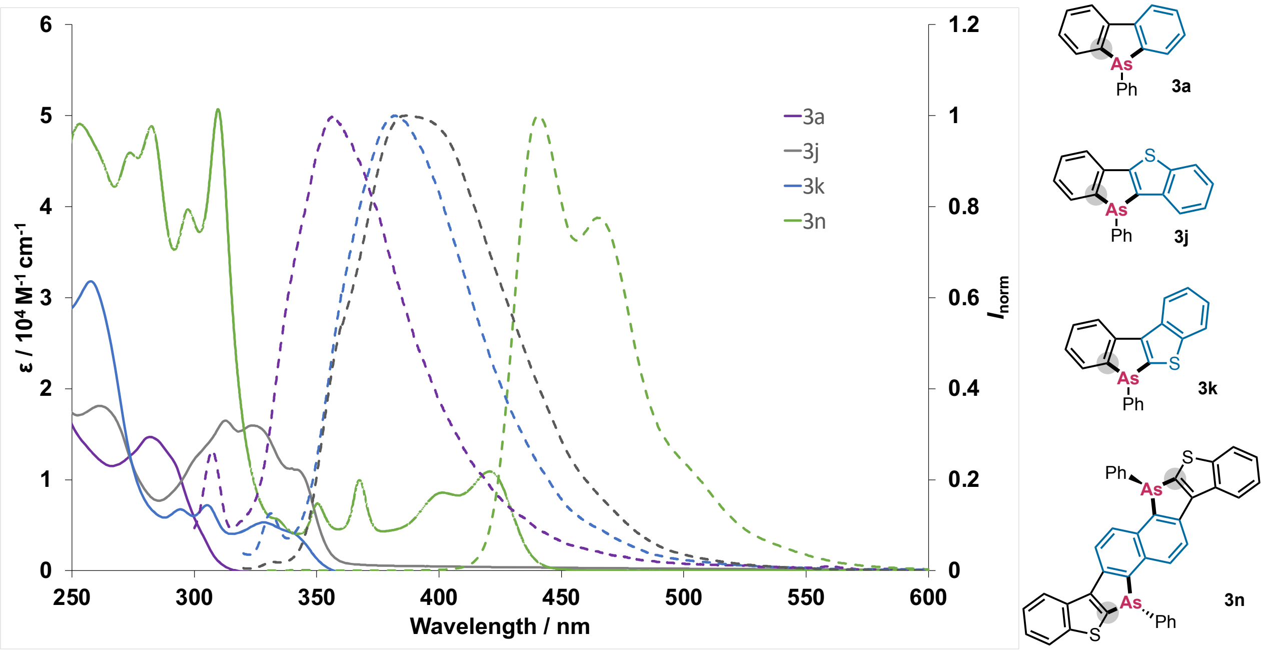

Figure S2. Absorption (solid lines) and normalized emission (dotted lines) spectra of **3a, 3j, 3k**, and **3n** (1.0×10^{-5} M in $CHCl_3$). Excited at 280 (**3a**), 300 (**3j**), 300 (**3k**), and 310 nm (**3n**), respectively.

Table S3. Summary of optical properties.

compound	λ_{abs} (nm) (ε (10^4 M^{-1} cm^{-1}))	λ_{Fl} (nm)	Φ (%)
**3a**	240 (2.4), 282 (1.5)	307, 357	1
**3j**	214 (1.5), 239 (2.4), 262 (1.8), 313 (1.6), 324 (1.6)	387	1
**3k**	240 (3.0), 258 (3.2), 294 (0.67), 305 (0.72), 328 (0.53)	331, 382	1
**3n**	253 (4.9), 274 (4.6), 282 (4.9), 297 (4.0), 310 (5.0), 350 (0.74), 367 (1.0), 402 (0.86), 421 (1.1)	441, 465	3

Electrochemical Properties

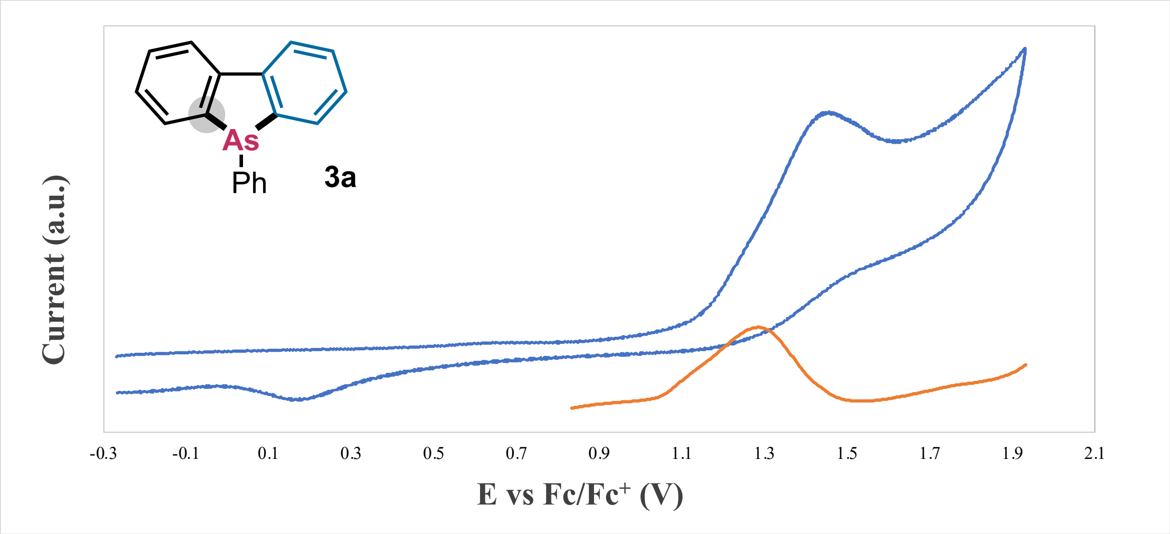

Figure S3. Cyclic voltammograms (CV, blue line, from 0 V to 2.0 V then back to 0 V) and differential pulse voltammograms (DPV, orange line) of **3a** in *o*-dichlorobenzene/acetonitrile (10:1, v/v) containing 0.1 M n-Bu_4NPF_6 at a scan rate of 0.10 Vs^{-1}.

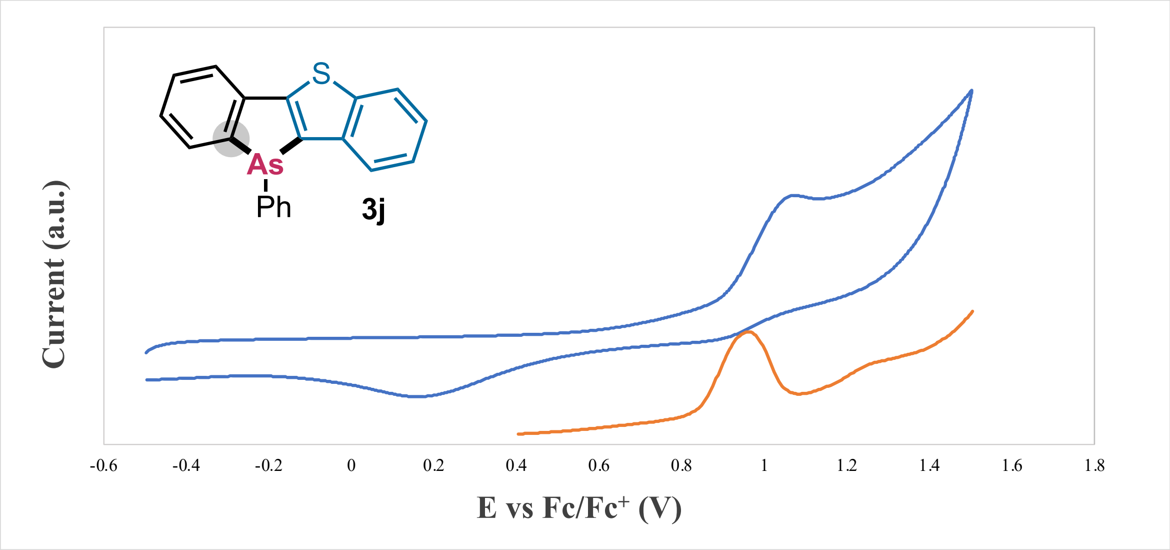

Figure S4. Cyclic voltammograms (CV, blue line, from 0 V to 2.0 V then back to 0 V) and differential pulse voltammograms (DPV, orange line) of **3j** in *o*-dichlorobenzene/acetonitrile (10:1, v/v) containing 0.1 M n-Bu_4NPF_6 at a scan rate of 0.050 Vs^{-1}.

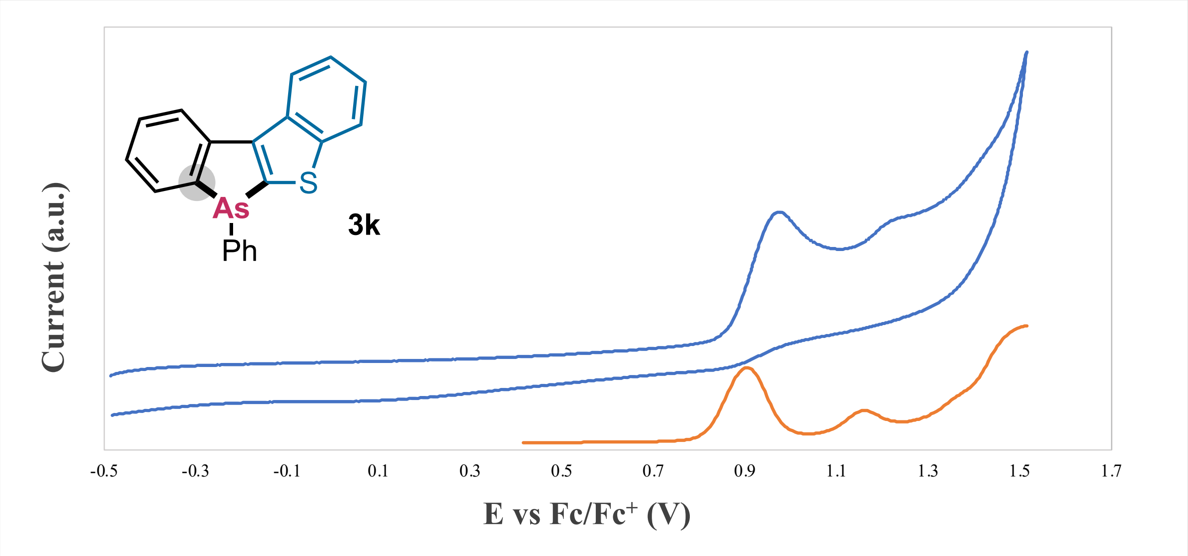

Figure S5. Cyclic voltammograms (CV, blue line, from 0 V to 2.0 V then back to 0 V) and differential pulse voltammograms (DPV, orange line) of **3k** in o-dichlorobenzene/acetonitrile (10:1, v/v) containing 0.1 M n-Bu_4NPF_6 at a scan rate of 0.050 Vs^{-1}.

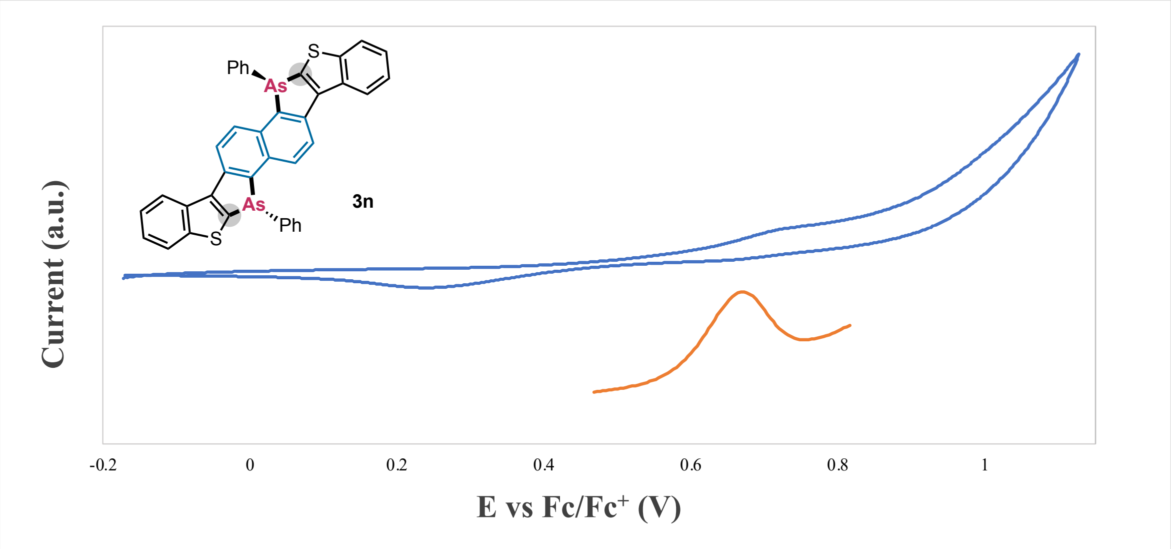

Figure S6. Cyclic voltammograms (CV, blue line, from 0 V to 1.5 V then back to 0 V) and differential pulse voltammograms (DPV, orange line) of **3n** in CH_2Cl_2 containing 0.1 M n-Bu_4NPF_6 at a scan rate of 0.030 Vs^{-1}.

Table S4. Summary of absorption wavelengths, HOMO-LUMO energy gaps and DPV of **3a**, **3j**, **3k**, and **3n**.

**Compd.**	λ_{onset}^{abs} (nm)[a]	E_g^{opt} (eV)[b]	$E^{1/2}_{ox}$ (V)[c]	E_{HOMO} (eV)[d]	E_{LUMO} (eV)[e]
**3a**	308	4.03	1.35	-6.15	-2.12
**3j**	357	3.47	0.96	-5.76	-2.29
**3k**	356	3.48	0.90	-5.70	-2.22
**3n**	441	2.81	0.67	-5.47	-2.66

[a] Measured in $CHCl_3$. [b] Determined from the onset of the normalized absorption spectra. [c] Performed in *o*-dichlorobenzene/MeCN (10:1, v/v, for **3a**, **3j**, and **3k**) or CH_2Cl_2 (for **3n**) in the presence of Bu_4NPF_6. v = 0.10 V/s (**3a**), 0.050 V/s (**3j** and **3k**), and 0.030 V/s (**3n**), versus Fc/Fc^+. [d] The approximation for Fc/Fc^+ level is -4.8 eV versus vacuum: $E_{HOMO} = -4.8 - E^{1/2}_{ox}$. [e] Estimated from E_{HOMO} and E_g^{opt}: $E_{LUMO} = E_{HOMO} + E_g^{opt}$.

References

(S1) (*a*) R. A. Batey and T. D. Quach, *Tetrahedron Lett.*, 2001, **42**, 9099. (*b*) W. Liu, P. Liu, L. Lv and C.-J. Li, *Angew. Chem., Int. Ed.*, 2018, **57**, 13499. (*c*) F. Rauch, P. Endres, A. Friedrich, D. Sieh, M. Hähnel, I. Krummenacher, H. Braunschweig, M. Finze, L. Ji and T. B. Marder, *Chem.–Eur. J.*, 2020, **26**, 12951.

(S2) (*a*) F. F. Blicke and F. D. Smith, *J. Am. Chem. Soc.*, 1929, **51**, 1558. (*b*) F. F. Blicke and F. D. Smith, *J. Am. Chem. Soc.*, 1929, **51**, 3479. (*c*) F. F. Blicke and E. L. Cataline, *J. Am. Chem. Soc.*, 1938, **60**, 419. (*d*) T. D. Sideris and P. V. Ioannou, *Phosphorus Sulfur Silicon Relat. Elem.*, 2006, **181**, 751. (*e*) A. M. Gregson, S. M. Wales, S. J. Bailey and P. A. Keller, *J. Organomet. Chem.*, 2015, **785**, 77.

(S3) T. Kato, S. Tanaka and K. Naka, *Chem. Lett.*, 2015, **44**, 1476.

(S4) A. Sumida, K. Naka and H. Imoto, *Chem.–Asian J.,* 2025, **20**, e202401767.

(S5) T. Fujii, A. Urushizuka, H. Imoto and K. Naka, *Asian J. Org. Chem.,* 2023, **2030**, e202300067.

(S6) V. I. Gavrilov, *Zhurnal Obshchei Khimii,* 1987, **57**, 347.

(S7) C. Takahara, S. Iwasaki, Y. Miyake, T. Yumura, H. Imoto and K. Naka, *Bull. Chem. Soc. Jpn.*, 2024, **97**, uoad028.

(S8) T. Igarashi, R. Shimazumi, N. Chatani and M. Tobisu, *Chem. Commun*., 2023, **59**, 9722.

[^{1}H and ^{13}C{^{1}H} NMR Spectra of **3a**]

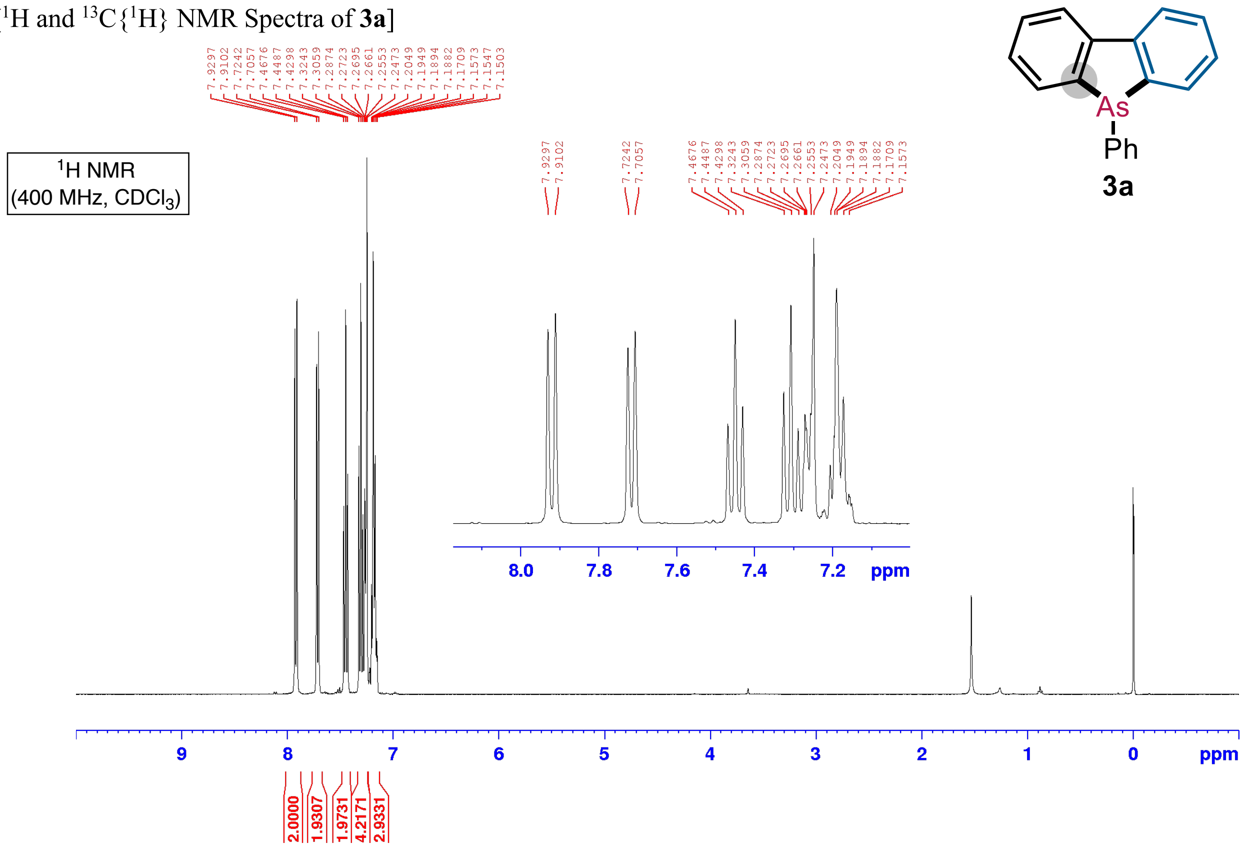

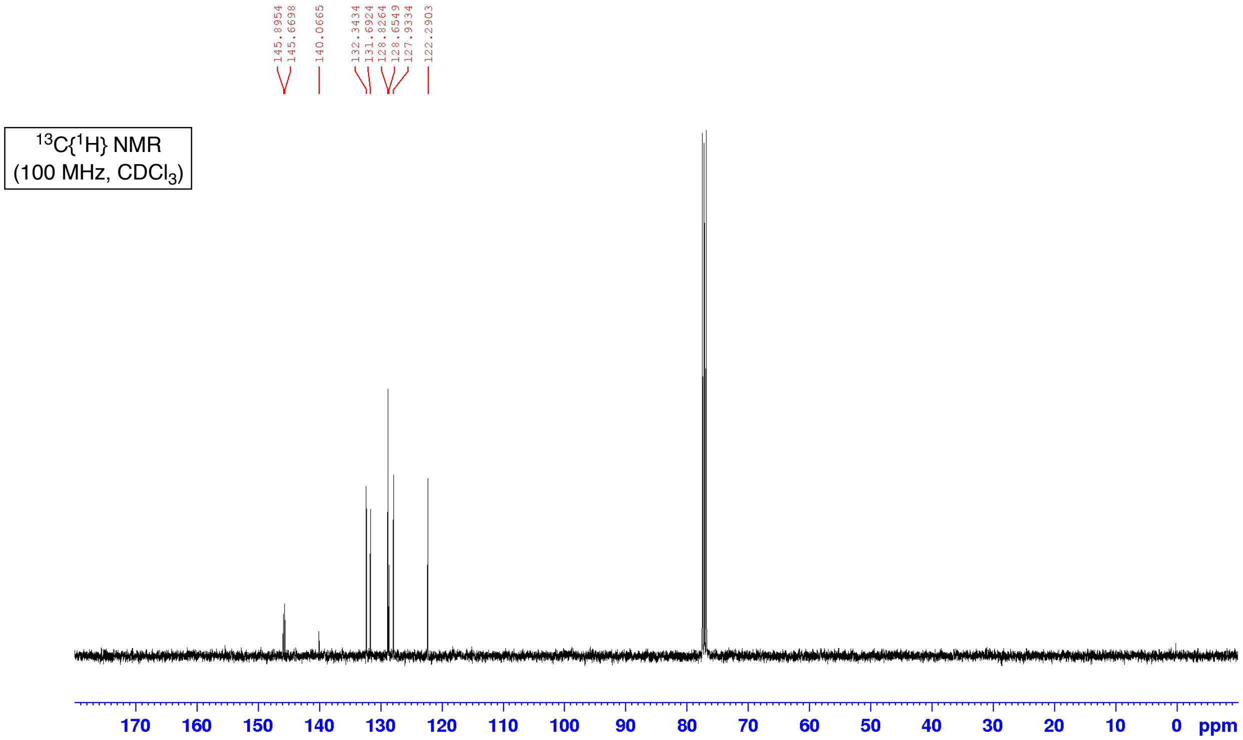

[^{1}H and ^{13}C{^{1}H} NMR Spectra of **3b**]

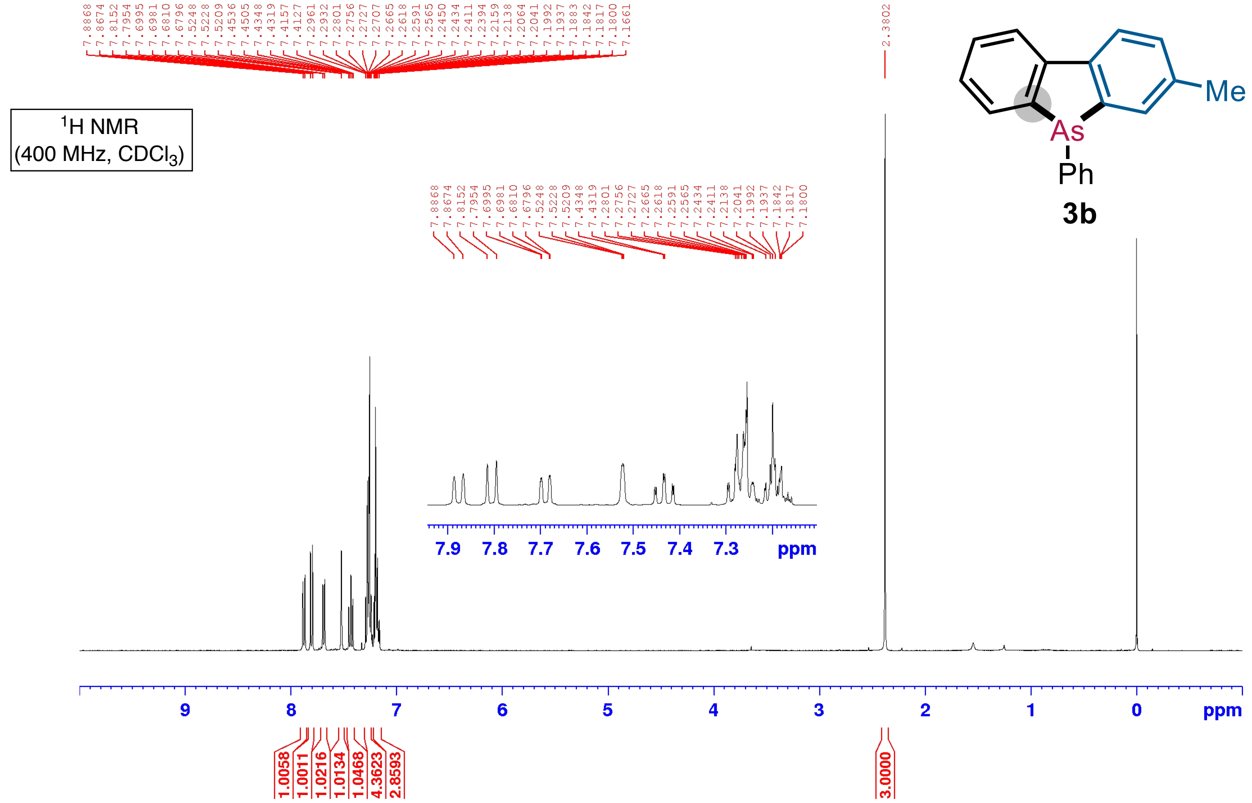

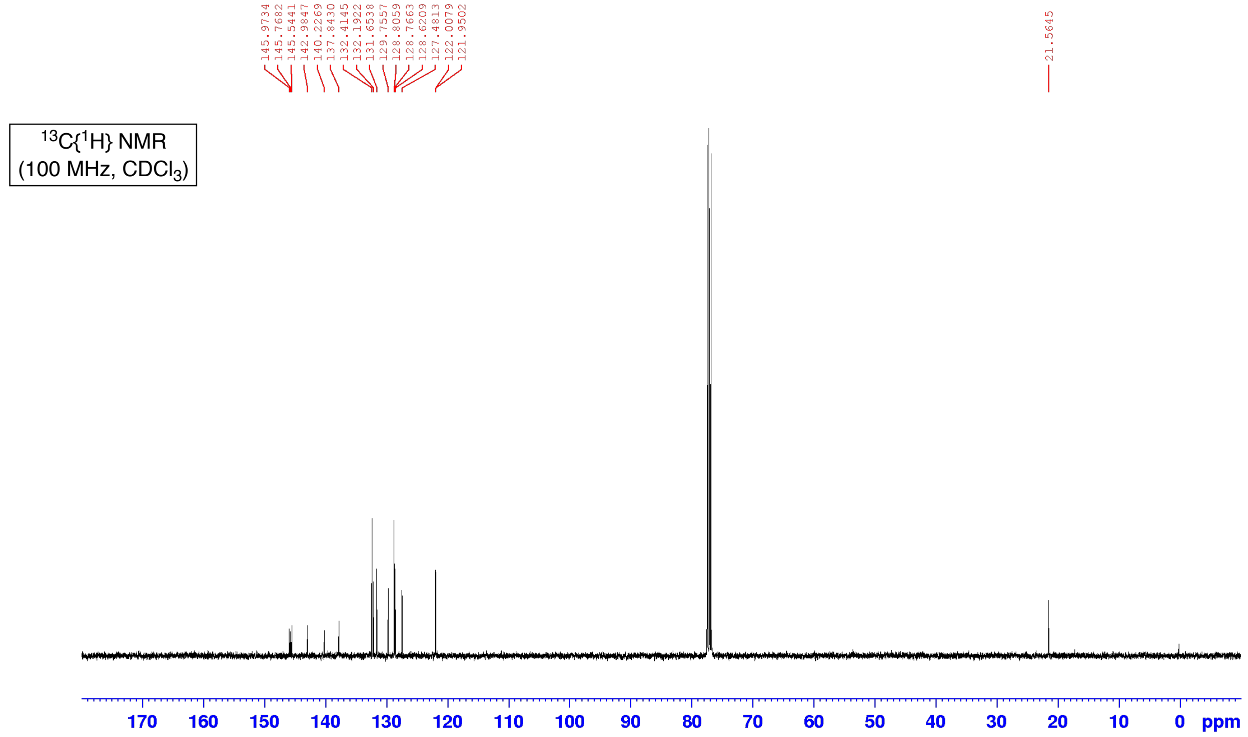

[^{1}H and ^{13}C{^{1}H} NMR Spectra of **3c**]

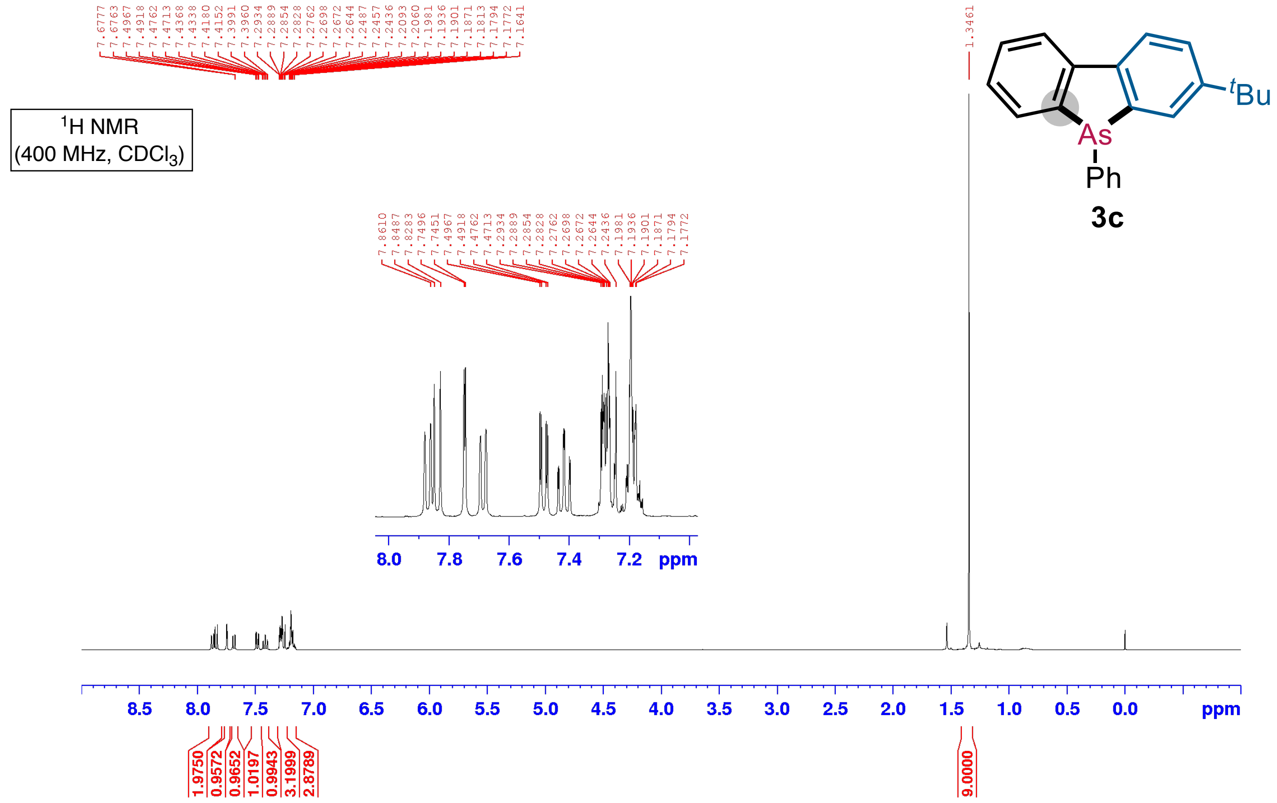

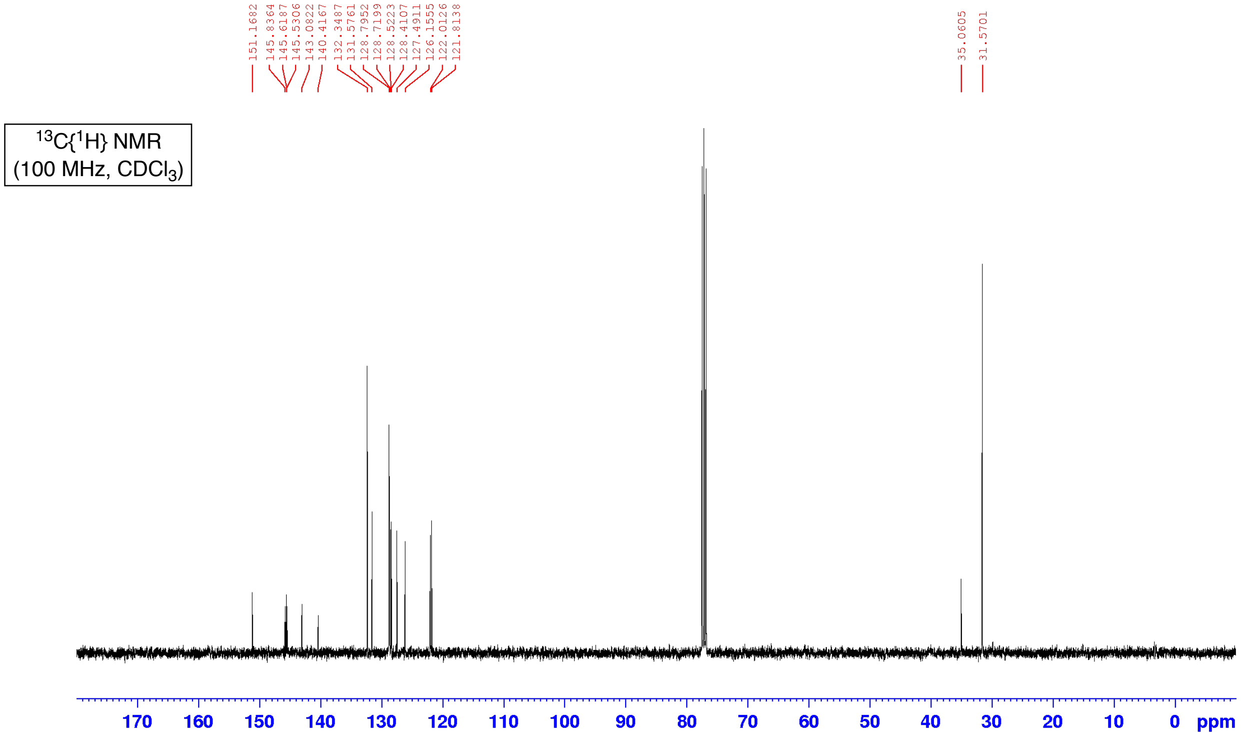

[^{1}H and ^{13}C{^{1}H} NMR Spectra of **3d**]

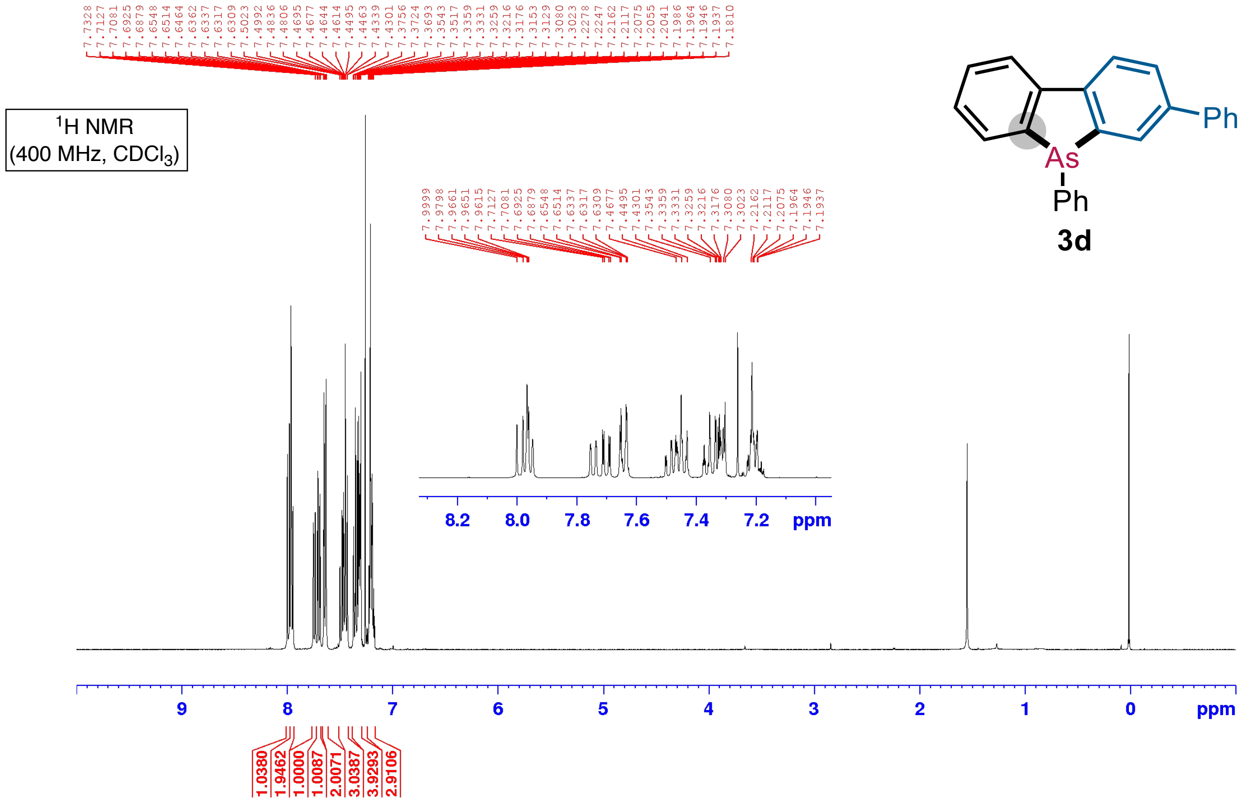

3d

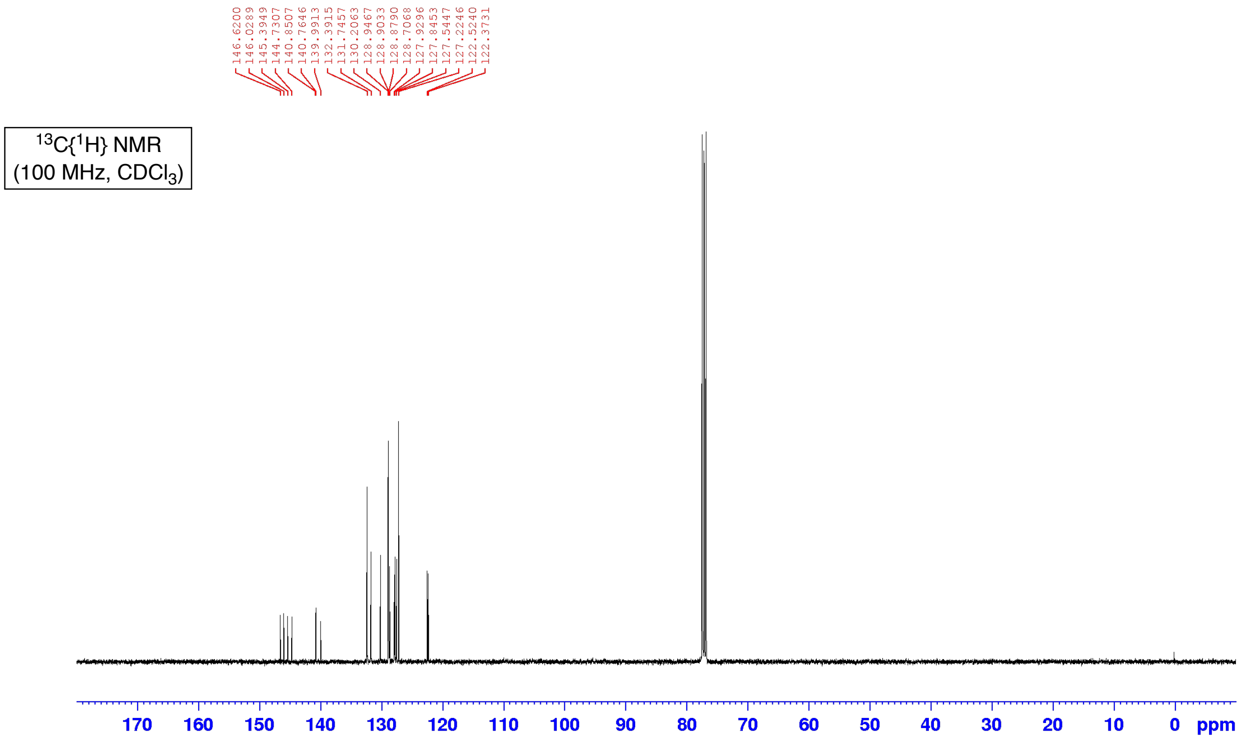

[^{1}H and ^{13}C{^{1}H} NMR Spectra of **3e**]

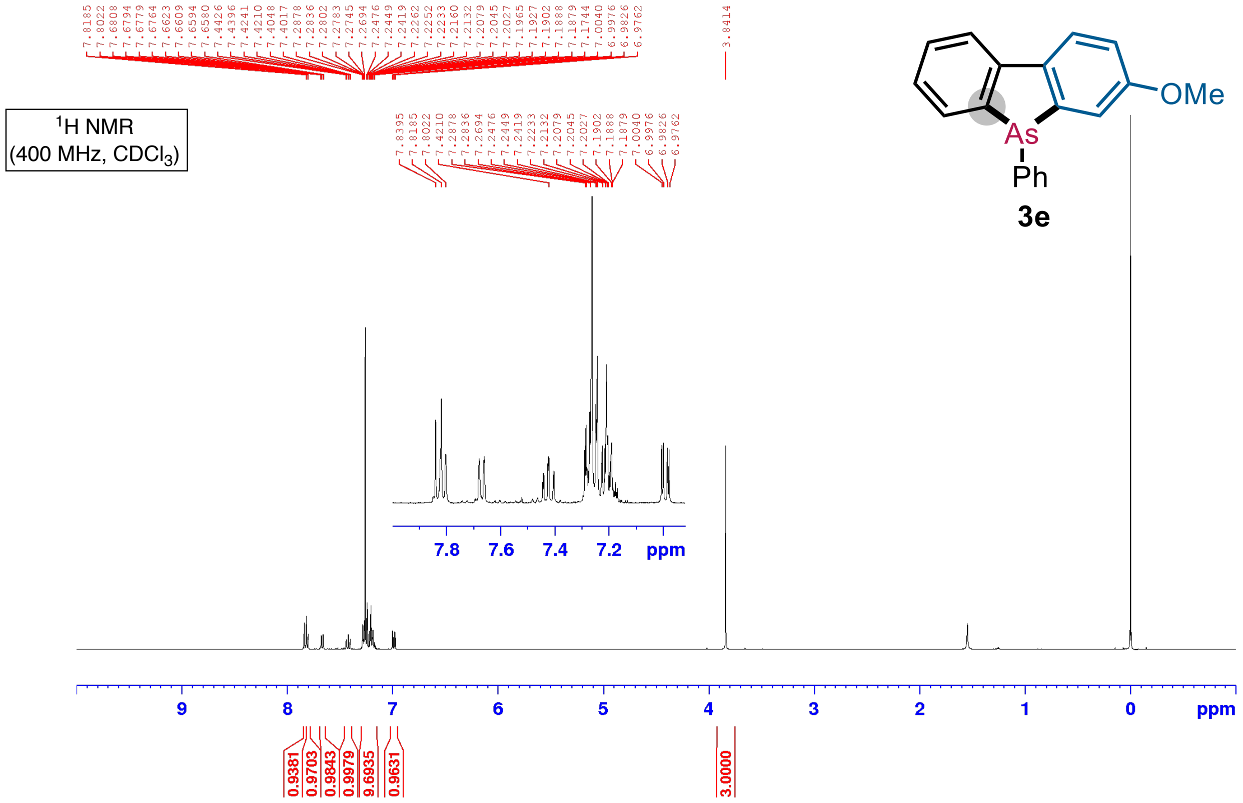

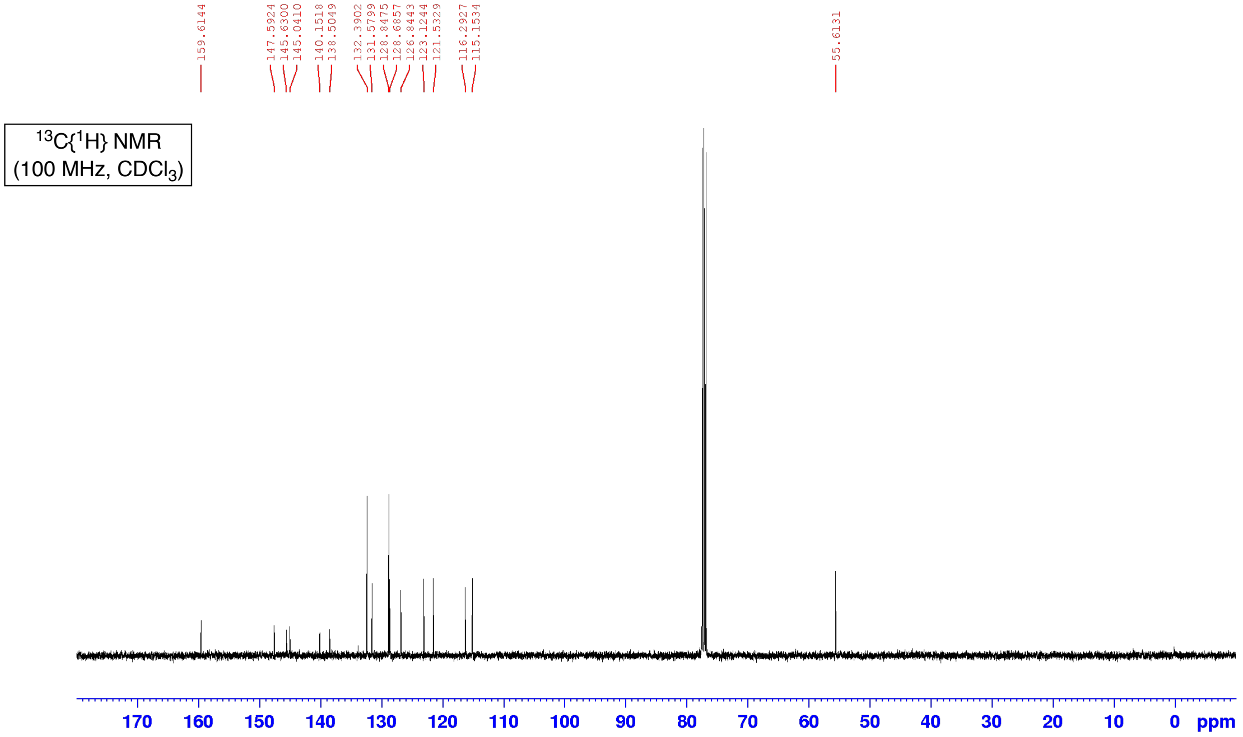

[^{1}H and ^{13}C{^{1}H} NMR Spectra of **3f**]

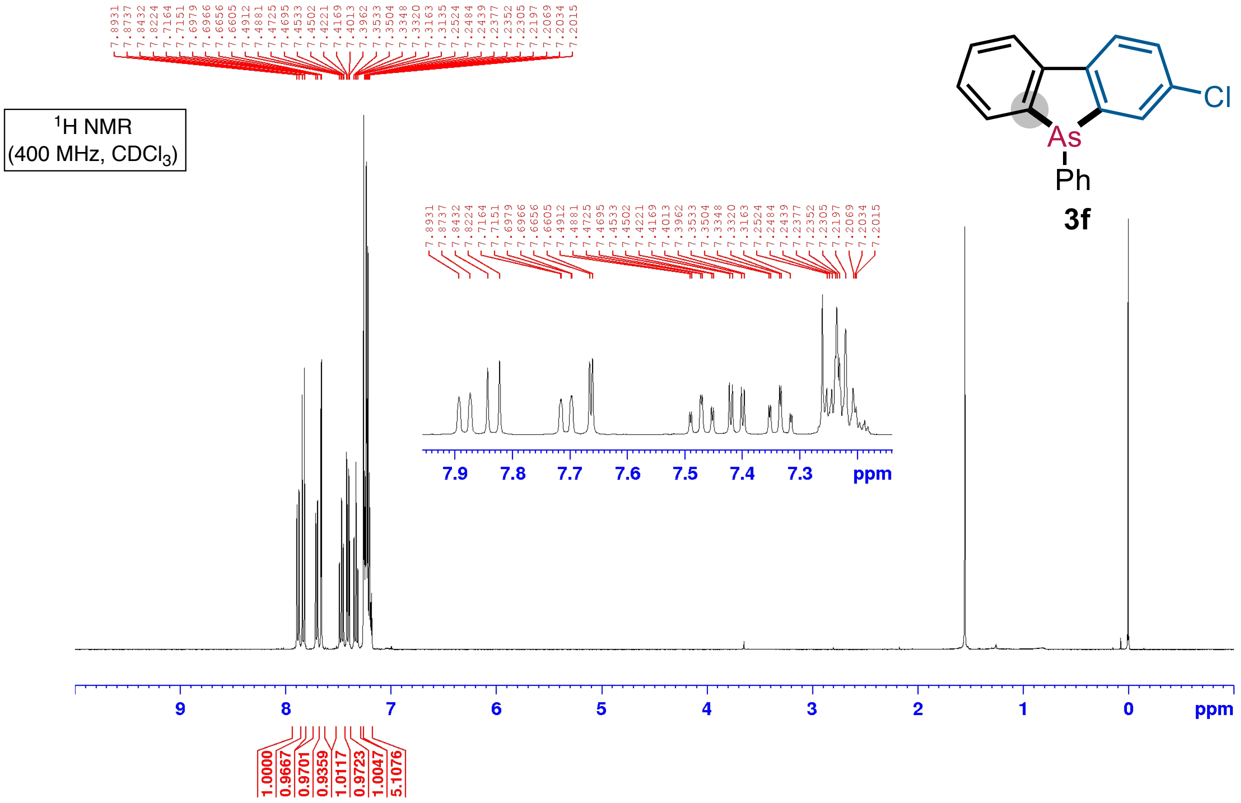

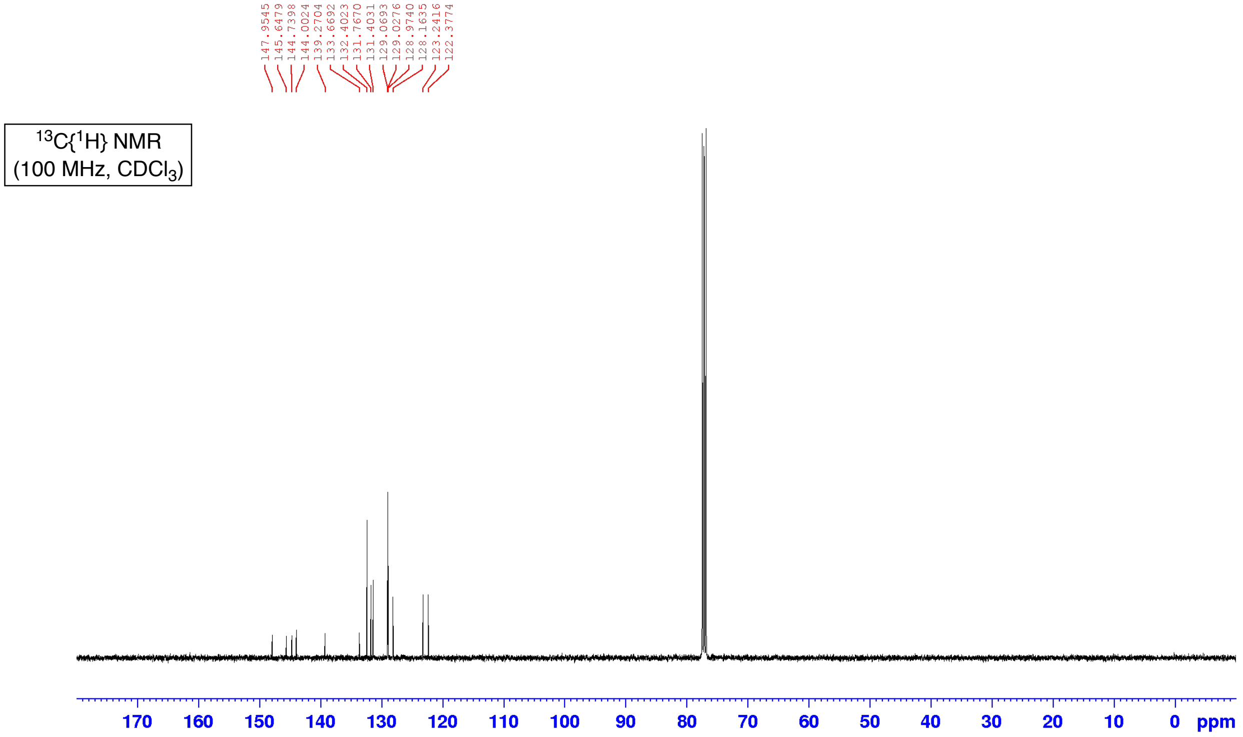

[^{1}H, ^{13}C{^{1}H}, and ^{19}F{^{1}H} NMR Spectra of **3g**]

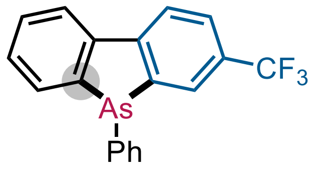

3g

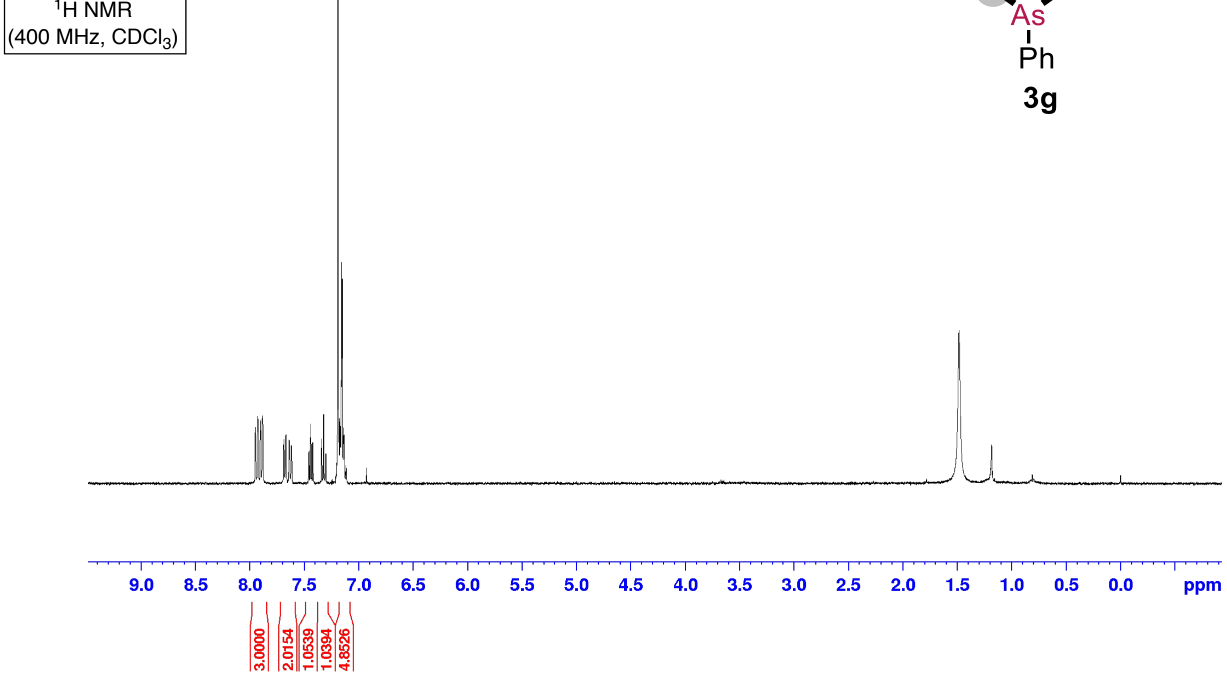

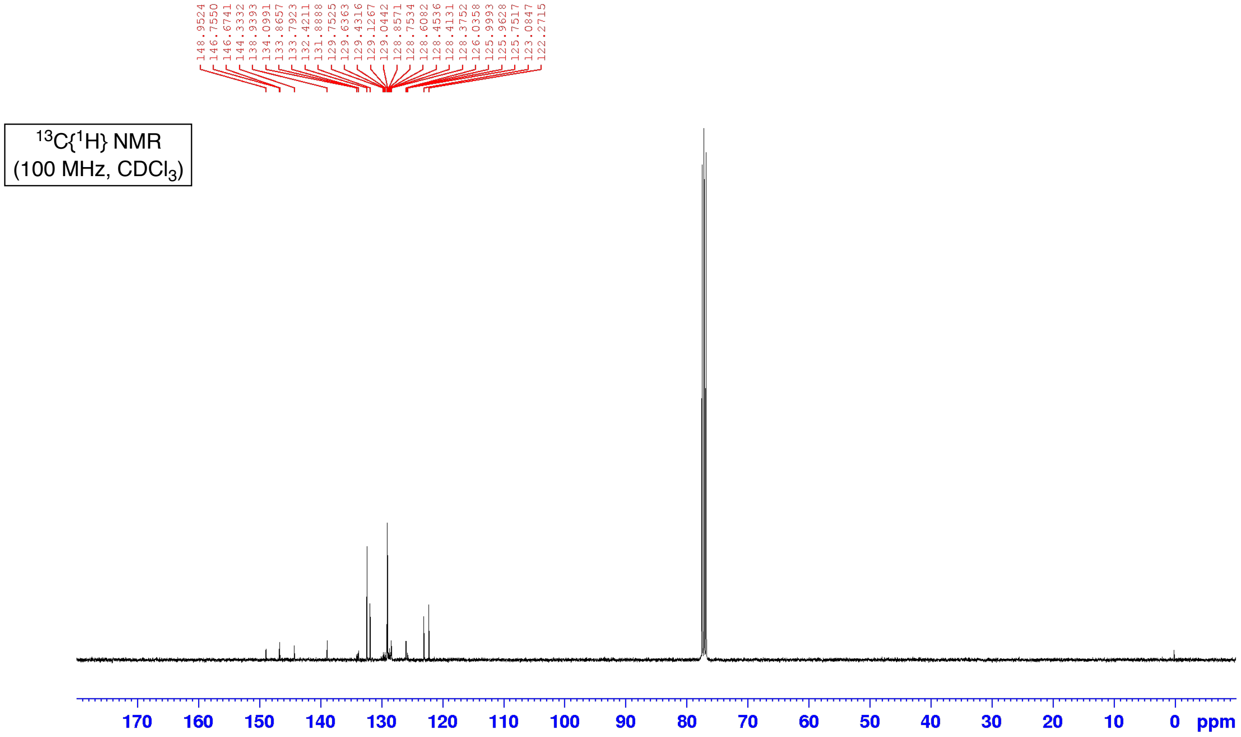

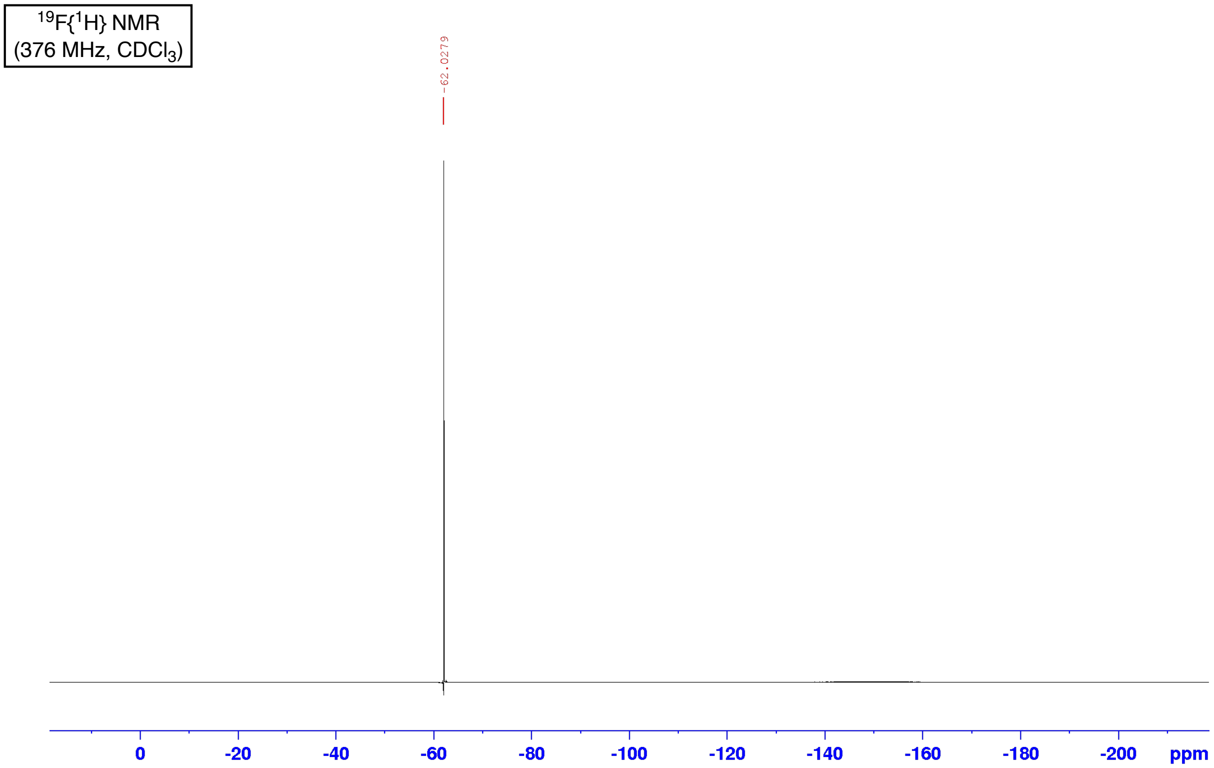

$^{19}F\{^1H\}$ NMR
(376 MHz, $CDCl_3$)
-62.0279
0
-20
-40
-60
-80
-100
-120
-140
-160
-180
-200
ppm

[^{1}H and ^{13}C{^{1}H} NMR Spectra of **3h**]

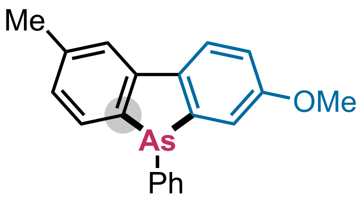

3h

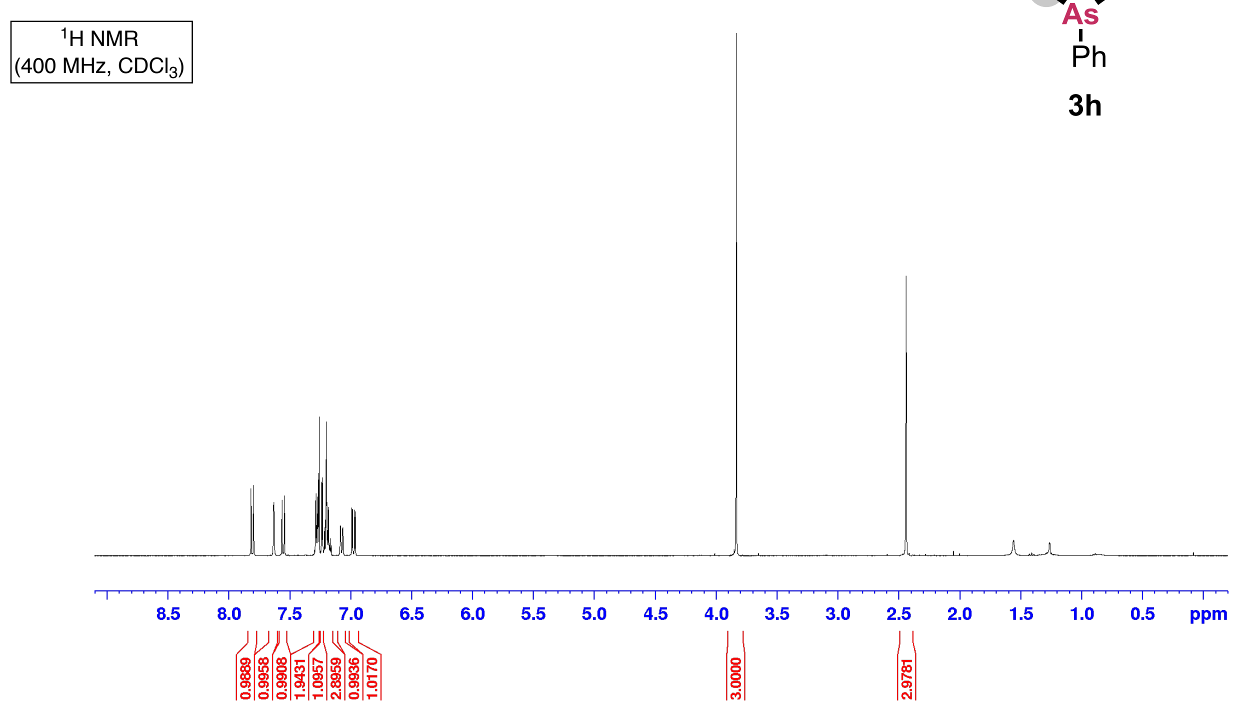

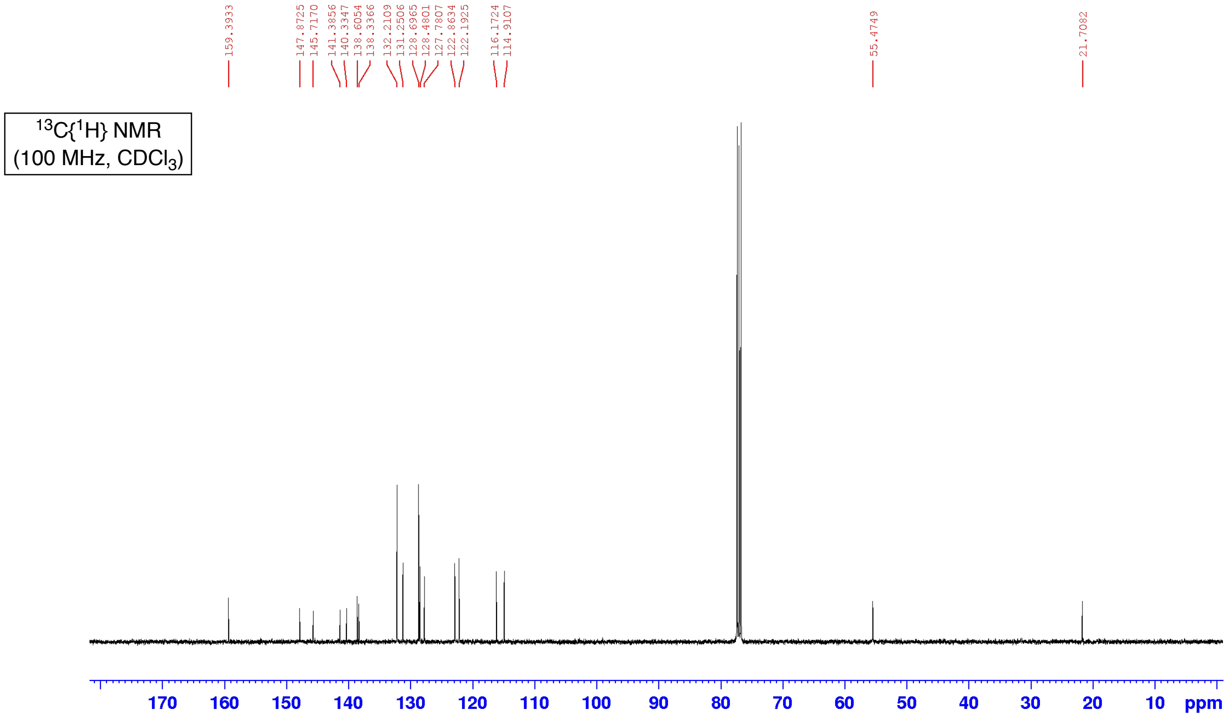

[^{1}H and ^{13}C{^{1}H} NMR Spectra of **3i**]

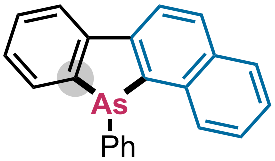

3i

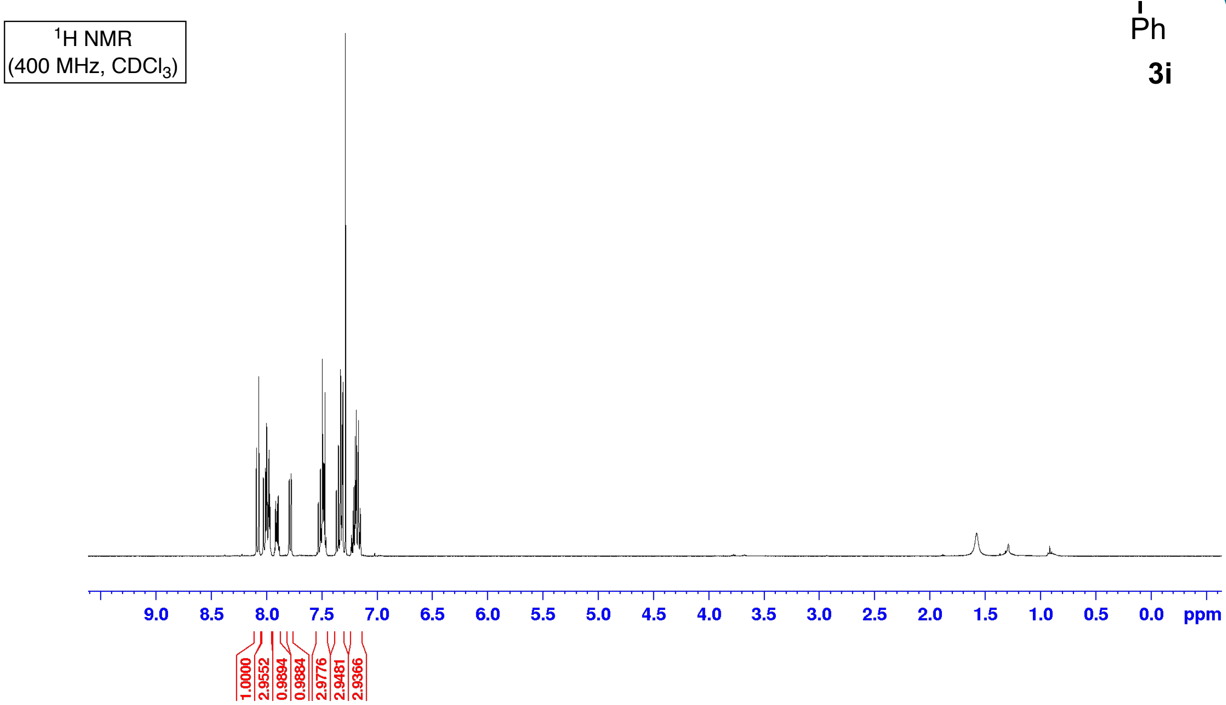

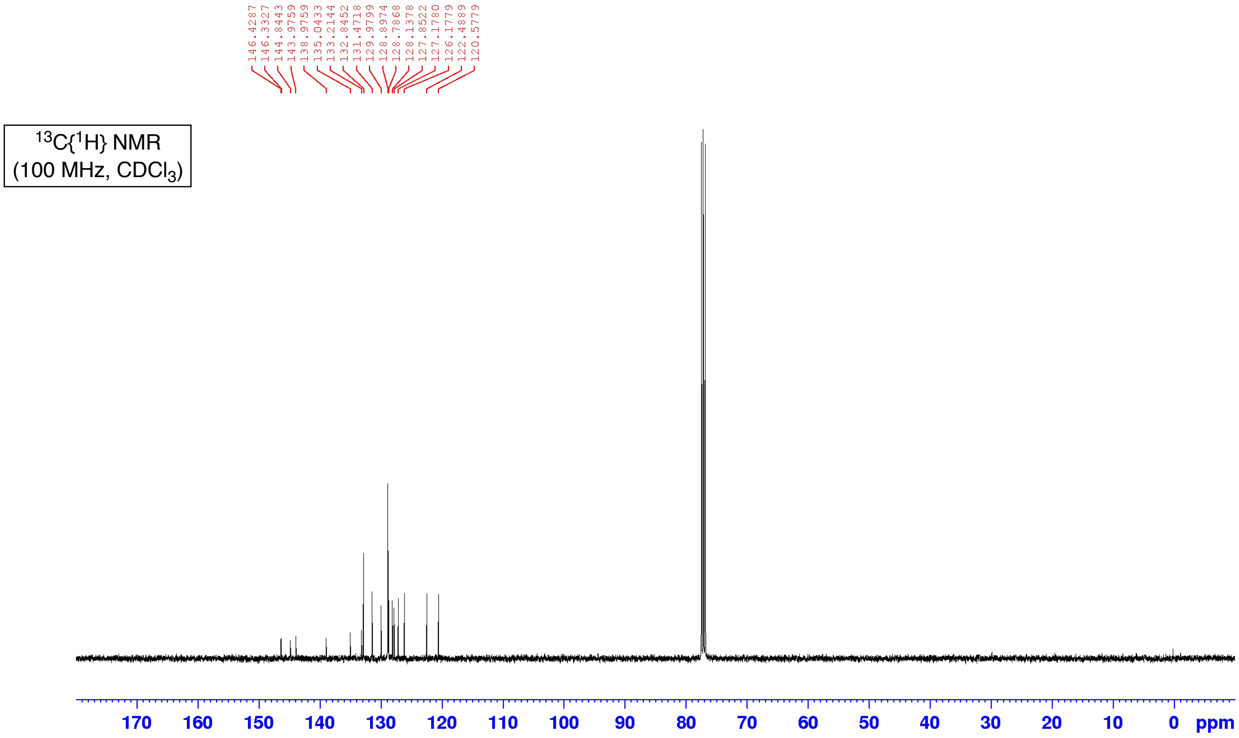

[^{1}H and ^{13}C{^{1}H} NMR Spectra of **3j**]

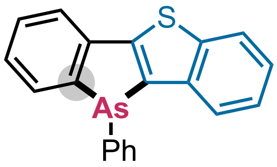

3j

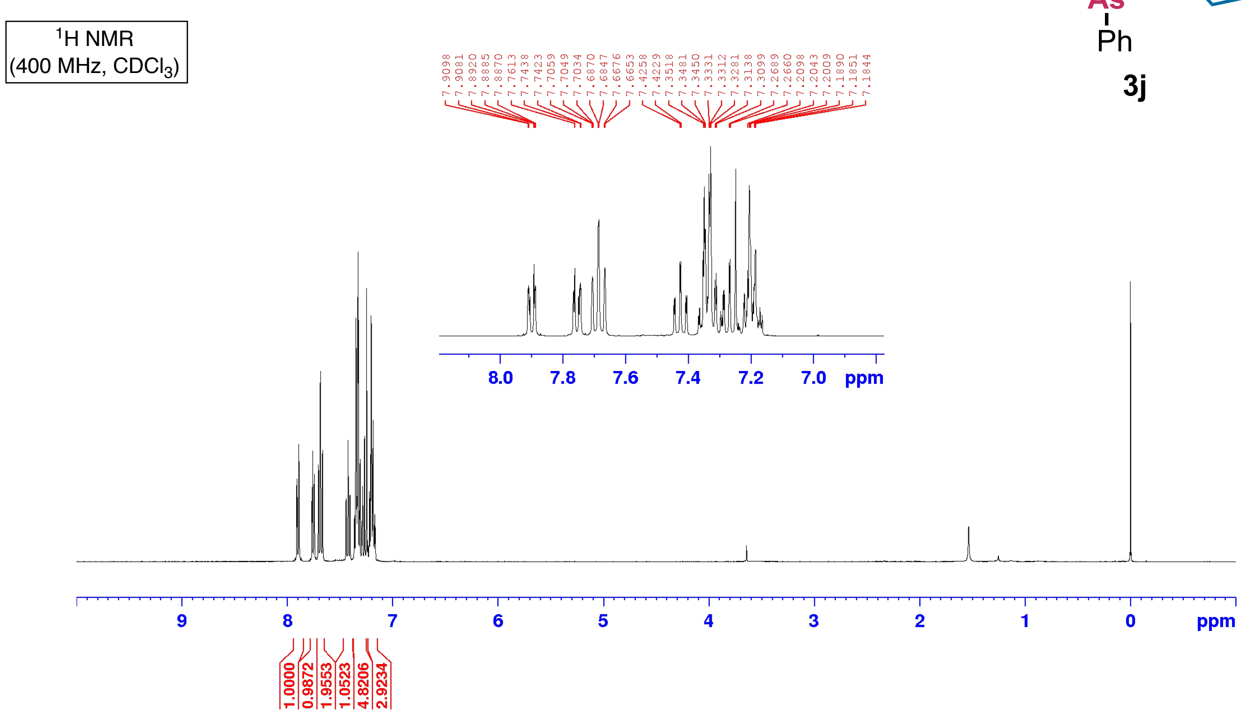

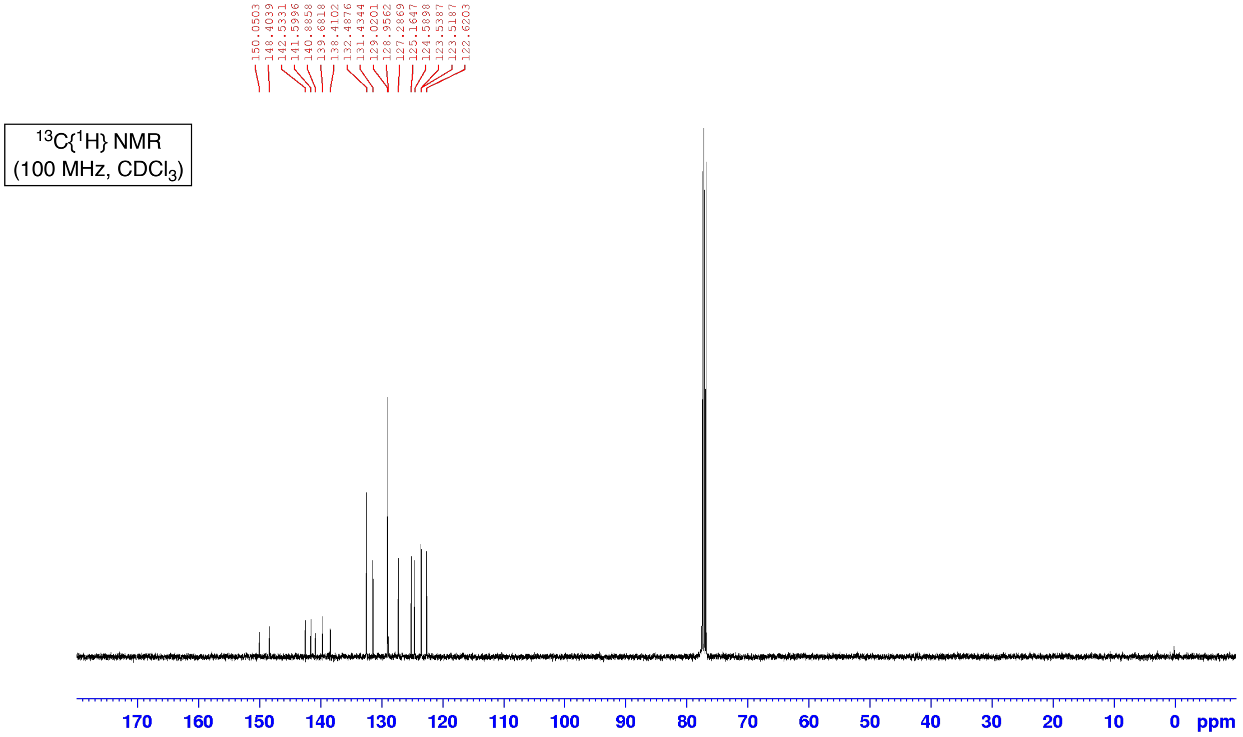

[^{1}H and ^{13}C{^{1}H} NMR Spectra of **3k**]

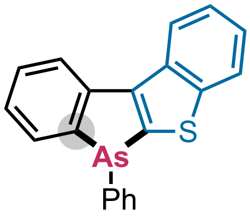

3k

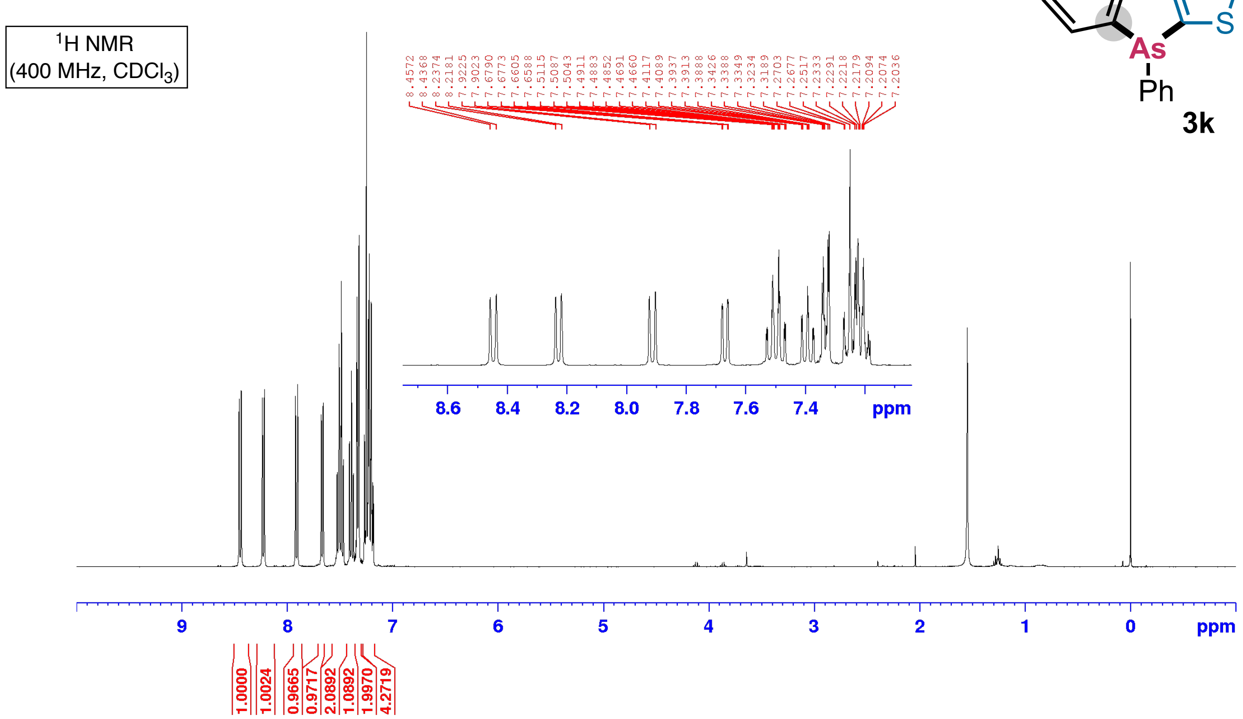

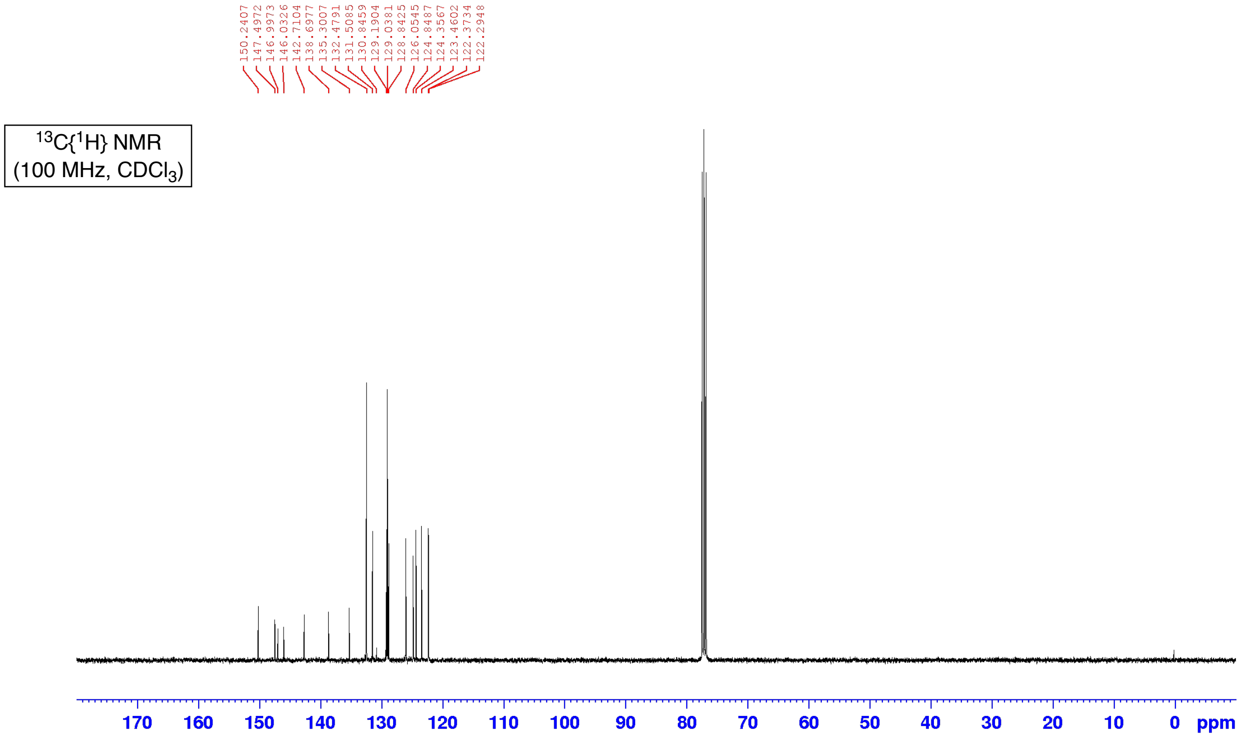

[^{1}H and $^{13}C\{^{1}H\}$ NMR Spectra of **3l**]

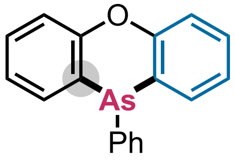

3l

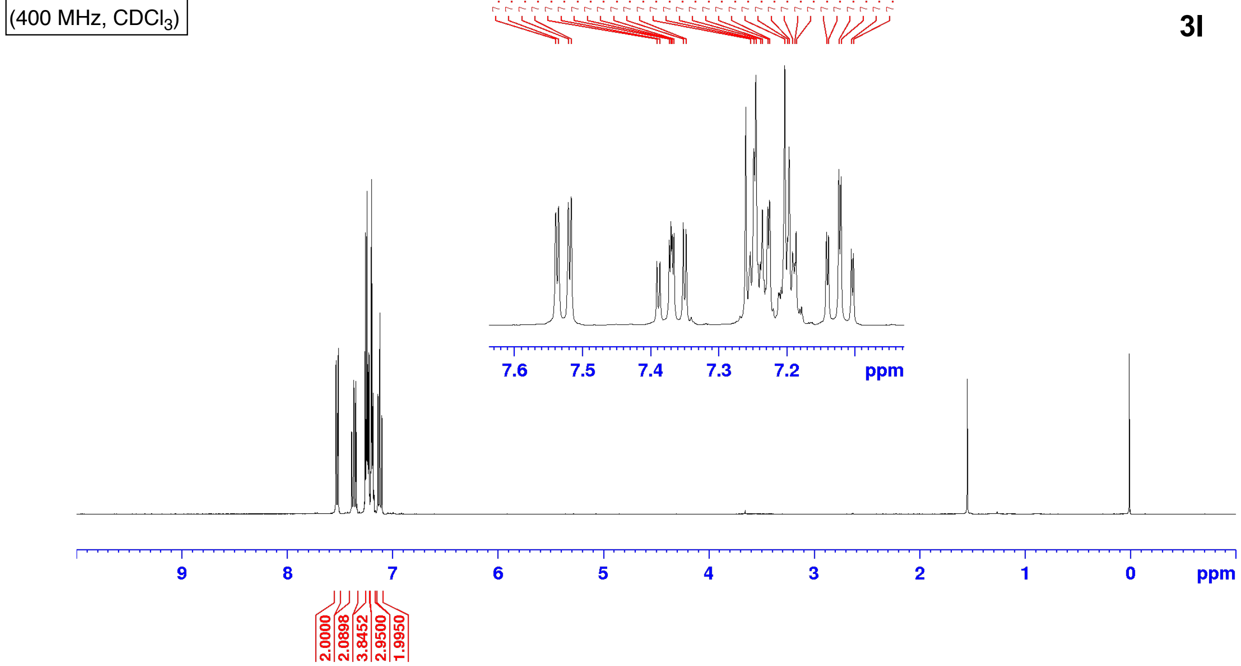

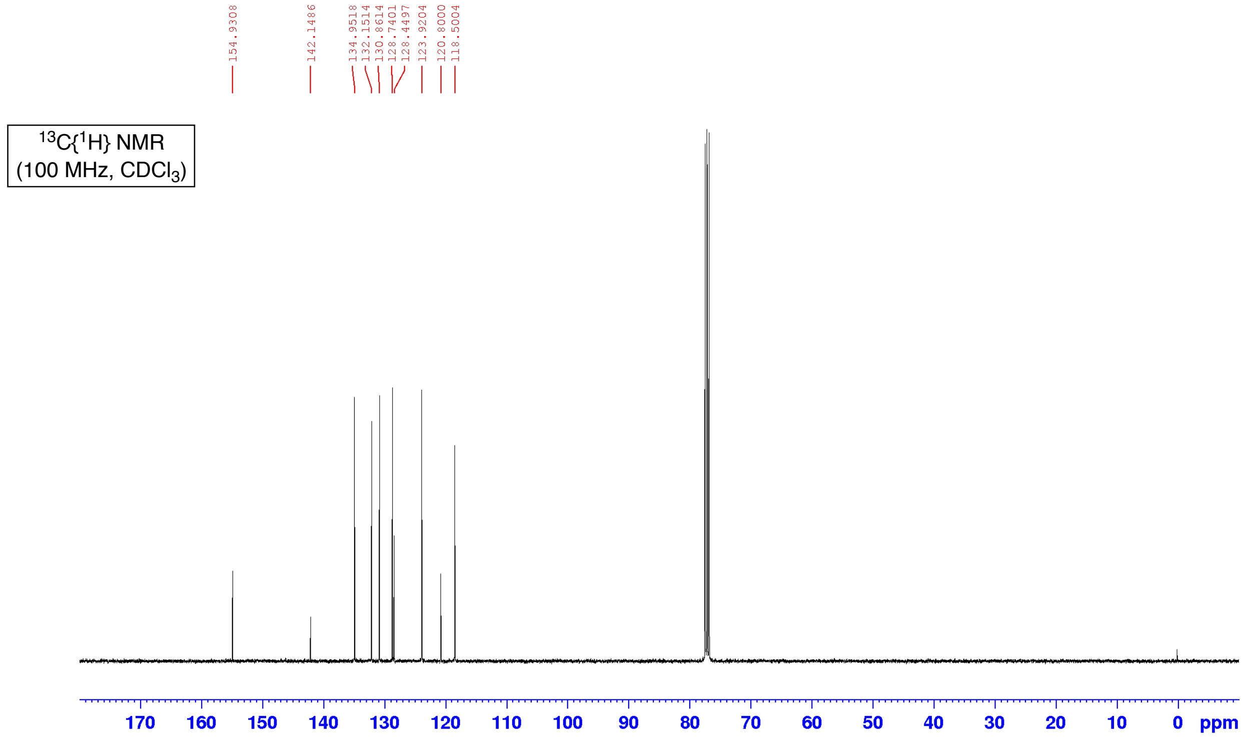

[^{1}H and ^{13}C{^{1}H} NMR Spectra of **3m**]

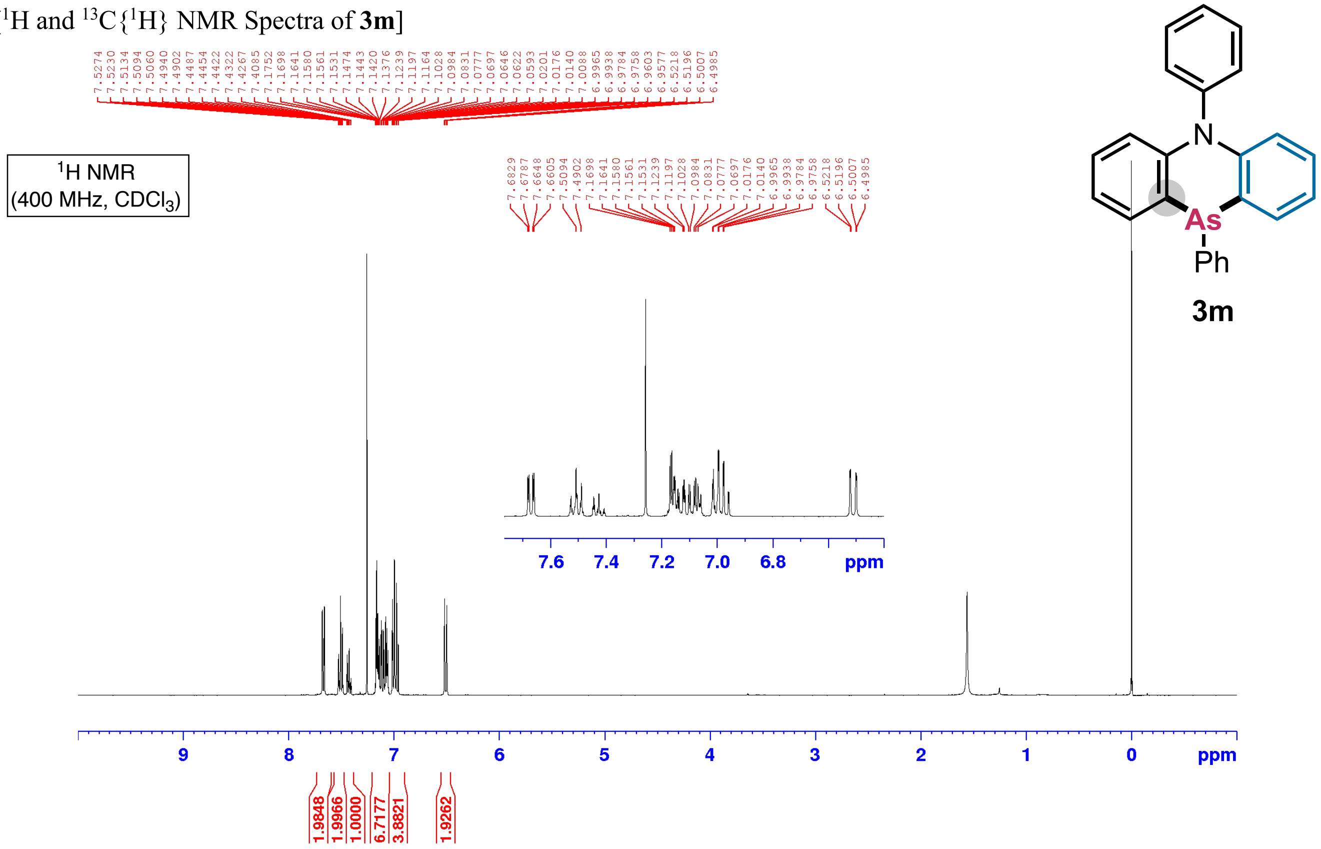

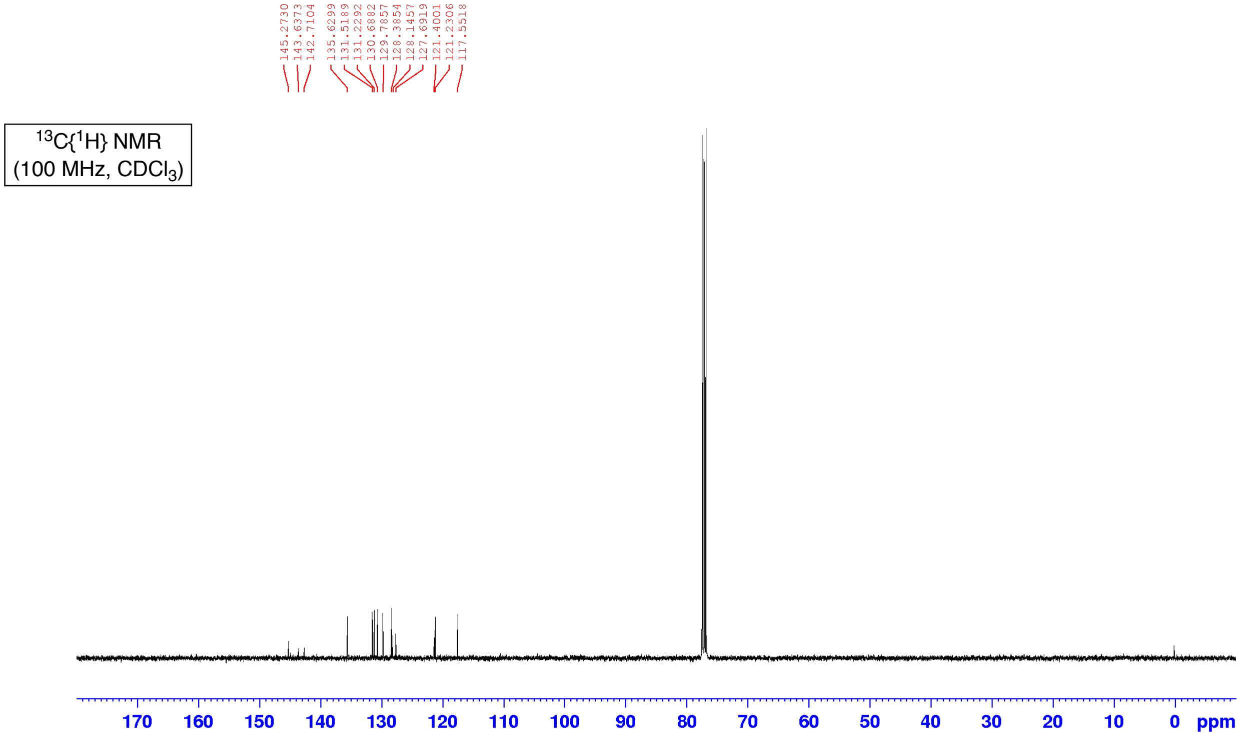

[^{1}H NMR Spectrum of **3n**]

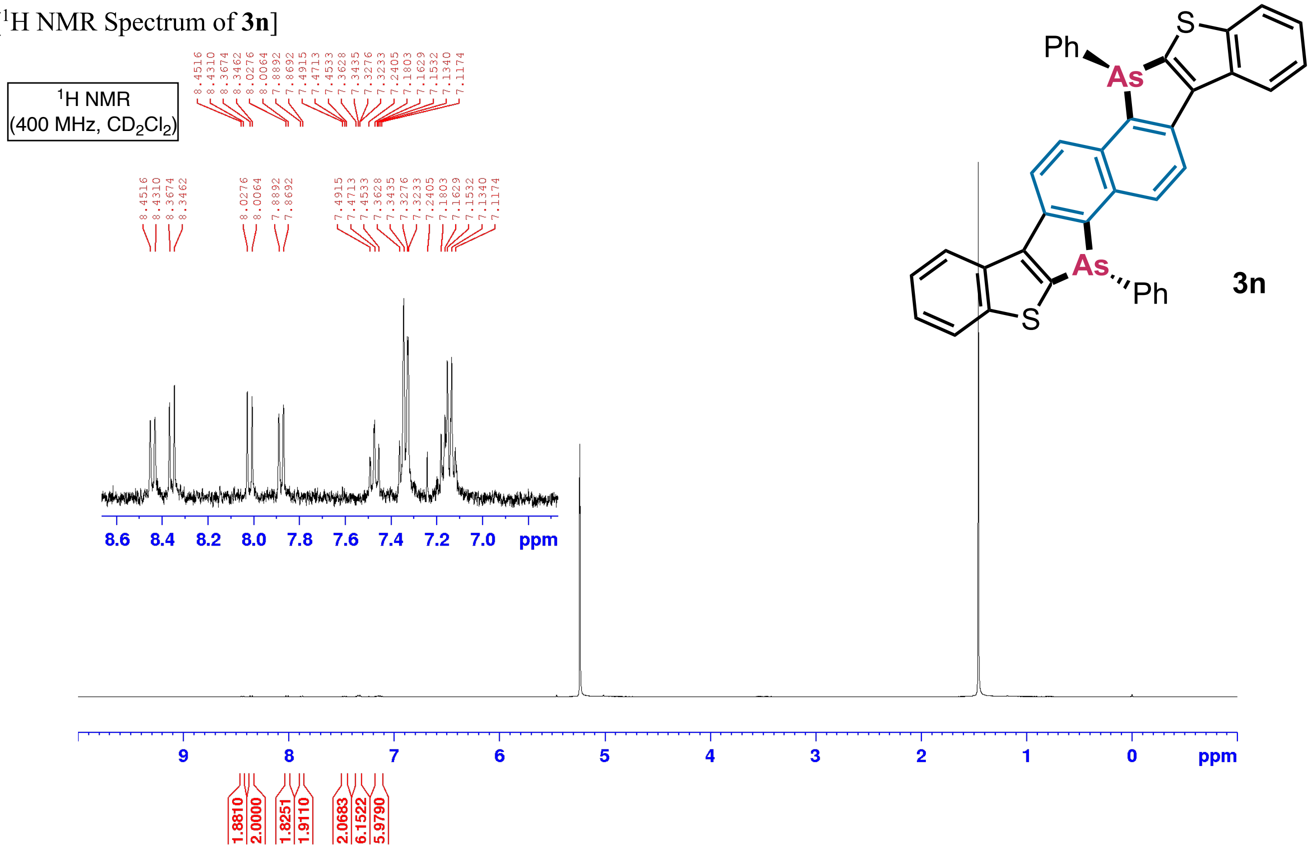

3n

[^{1}H and ^{13}C{^{1}H} NMR Spectra of **4a**]

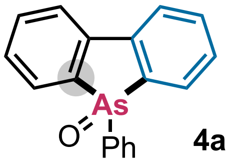

4a

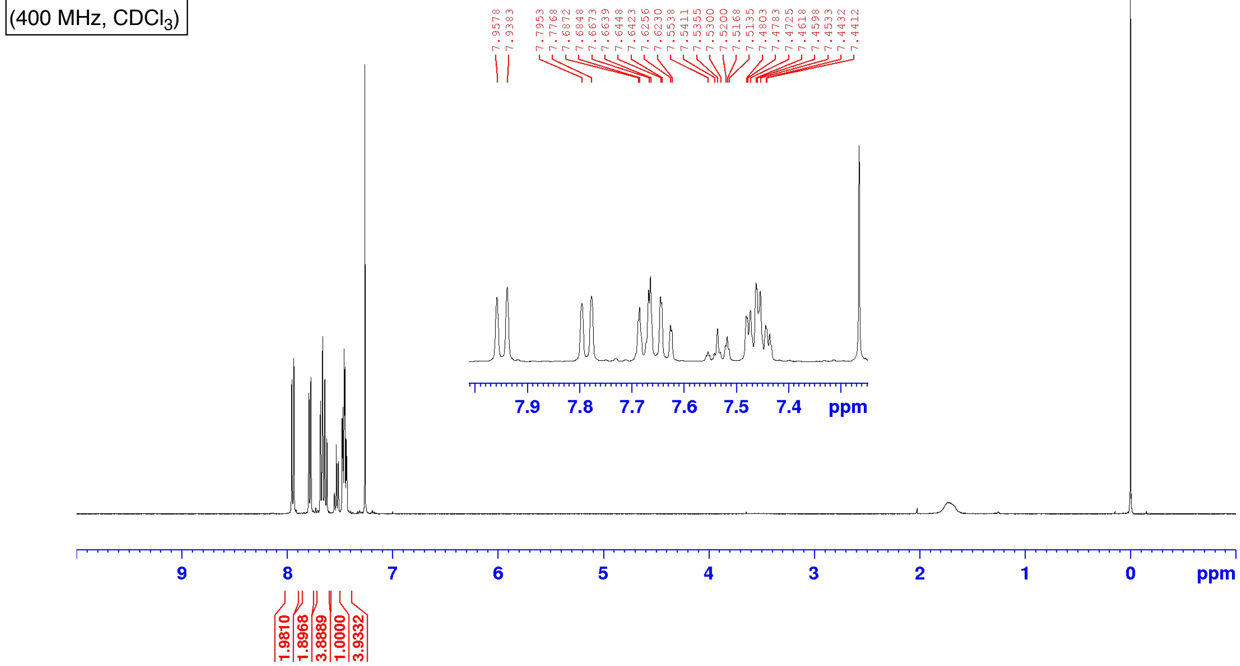

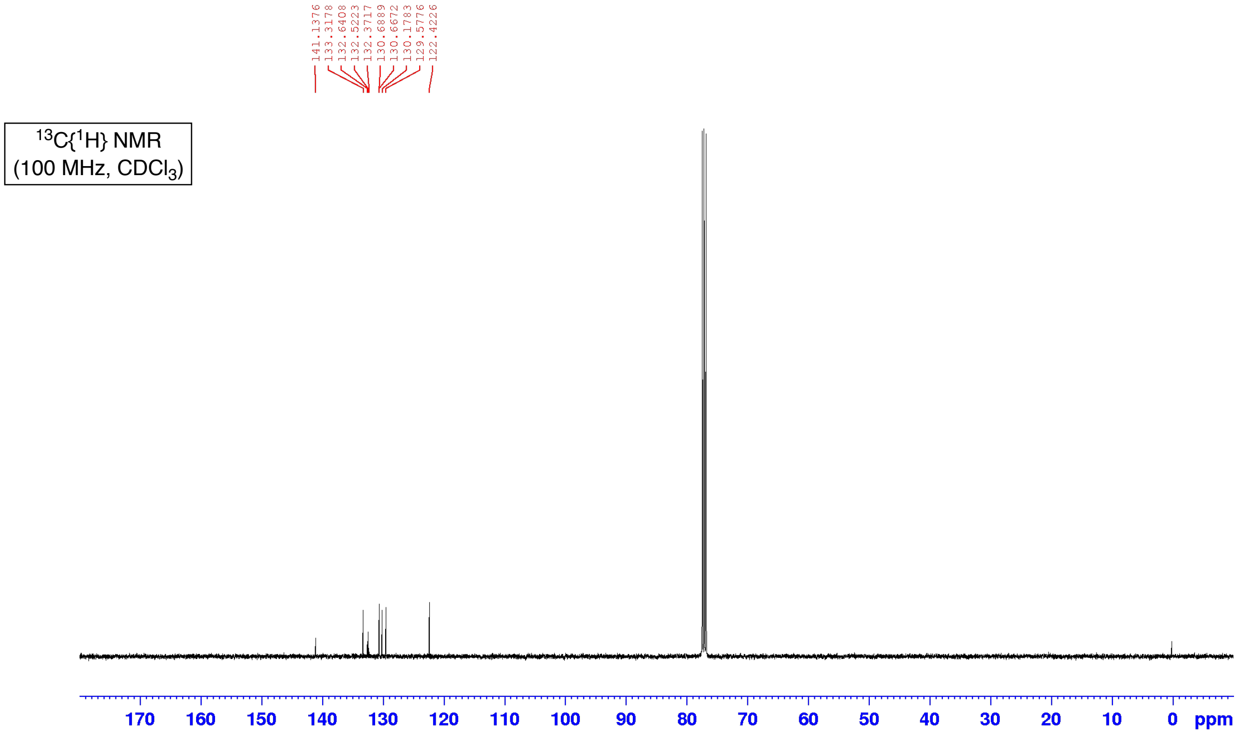

[^{1}H and ^{13}C{^{1}H} NMR Spectra of **5a**]

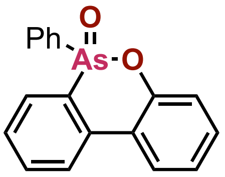

5a

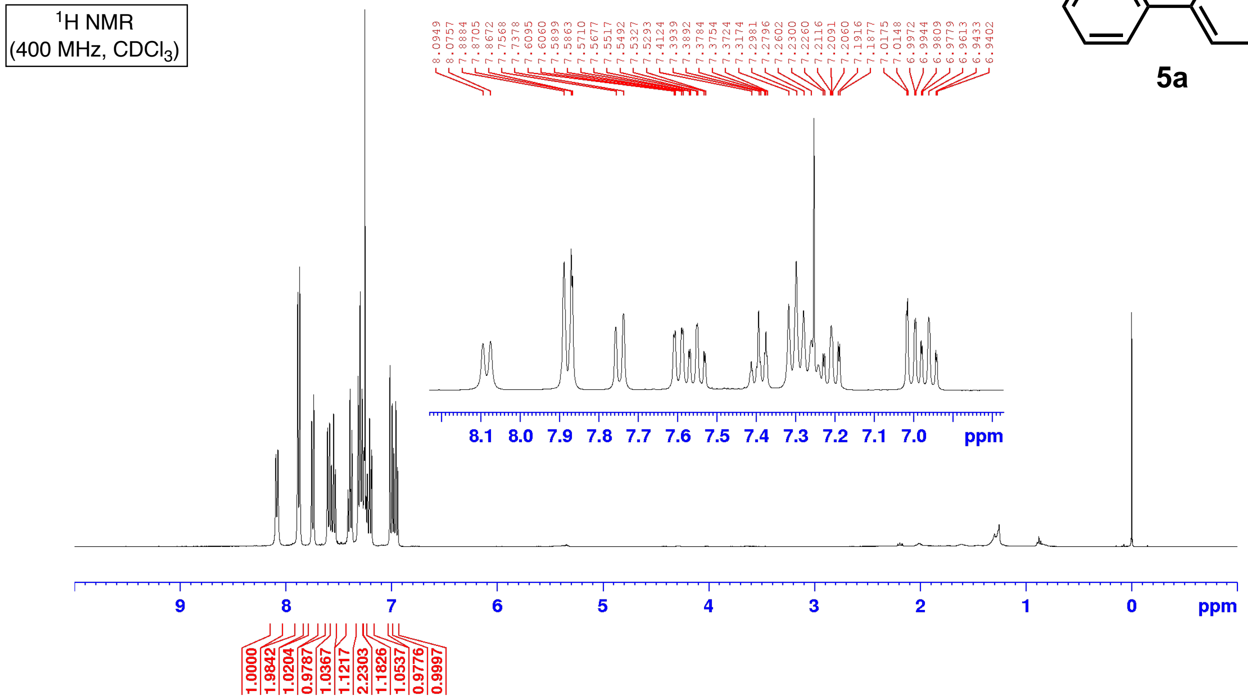

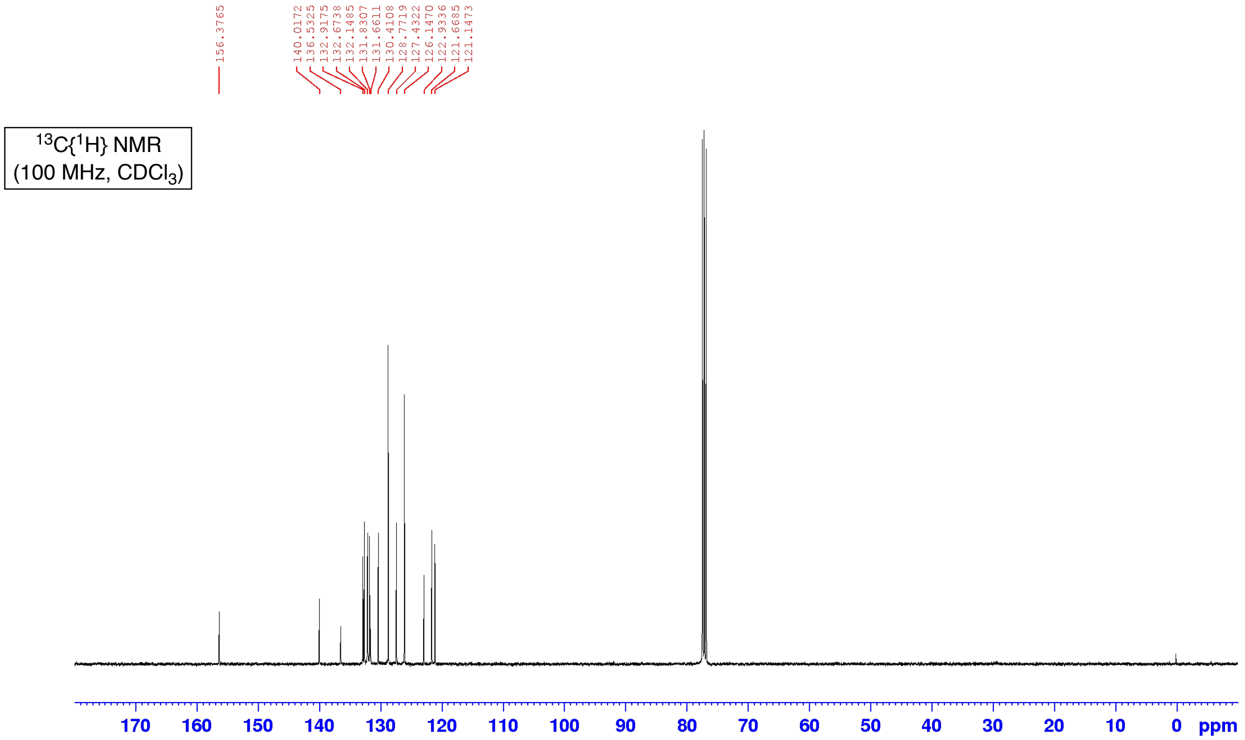

[^{1}H and ^{13}C{^{1}H} NMR Spectra of **5b**]

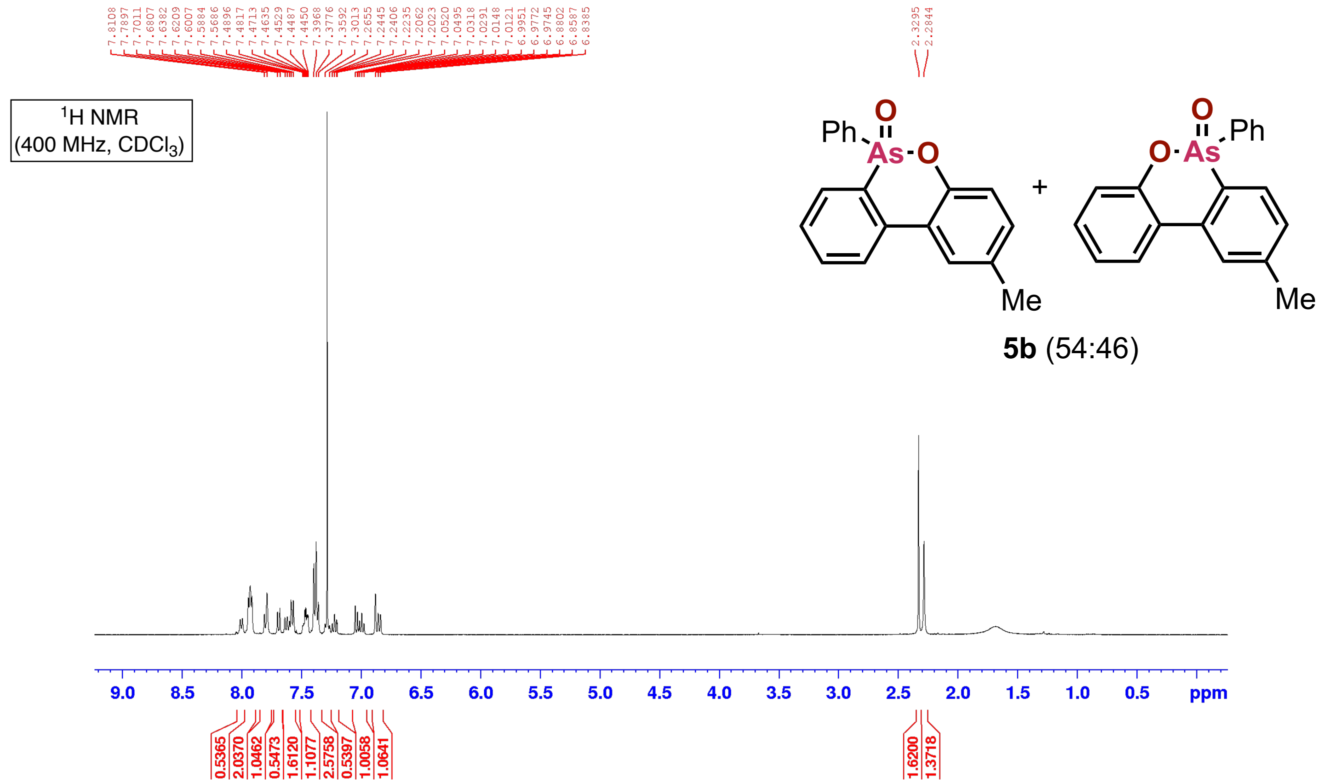

5b (54:46)

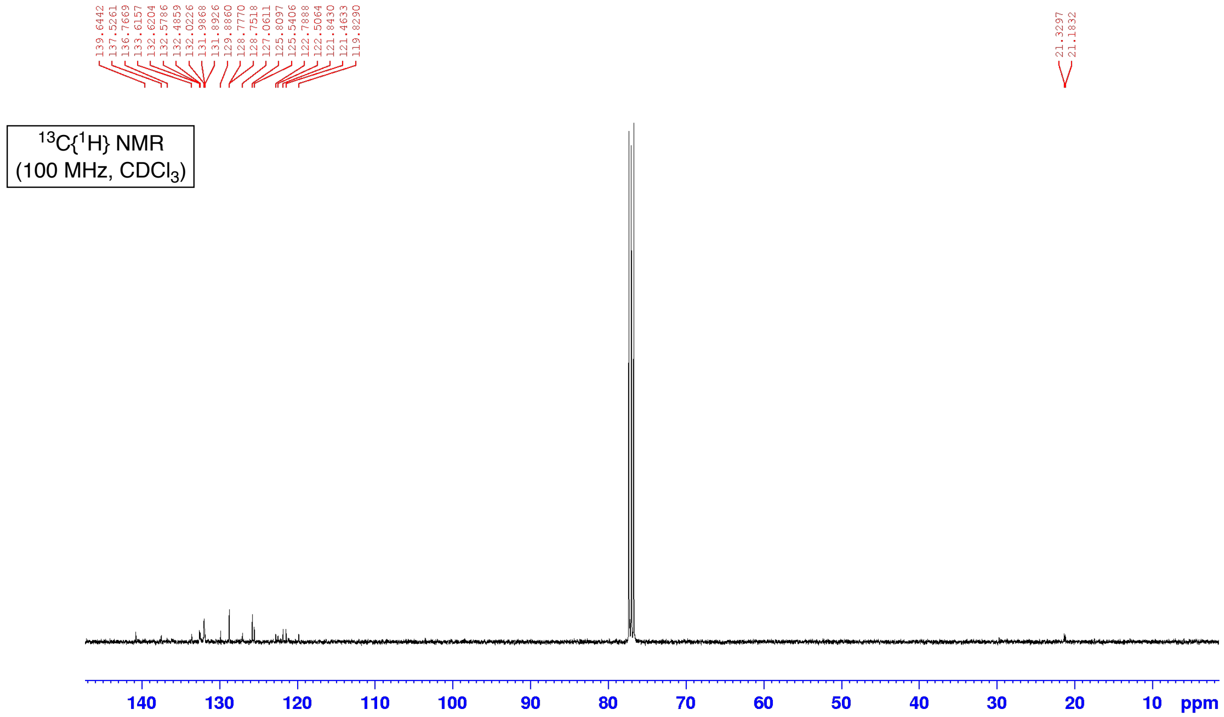

[^{1}H and ^{13}C{^{1}H} NMR Spectra of **6a**]

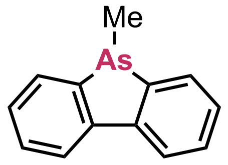

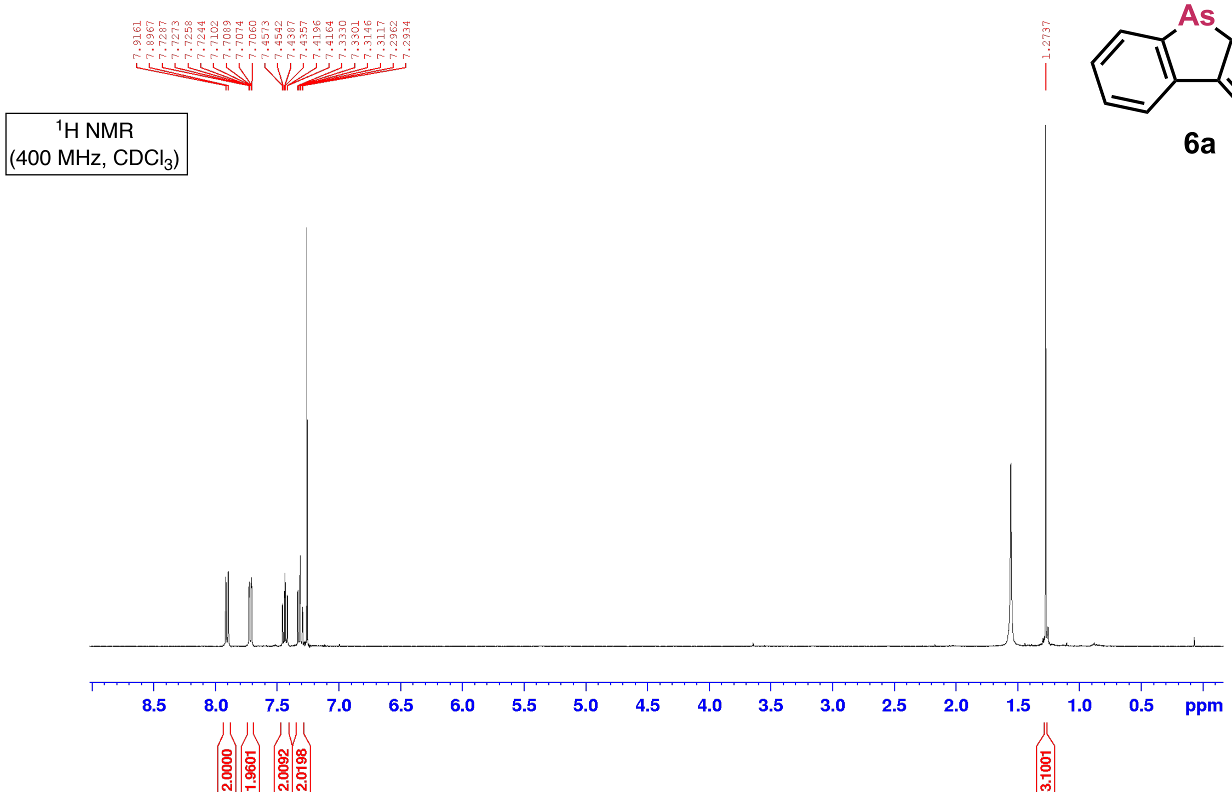

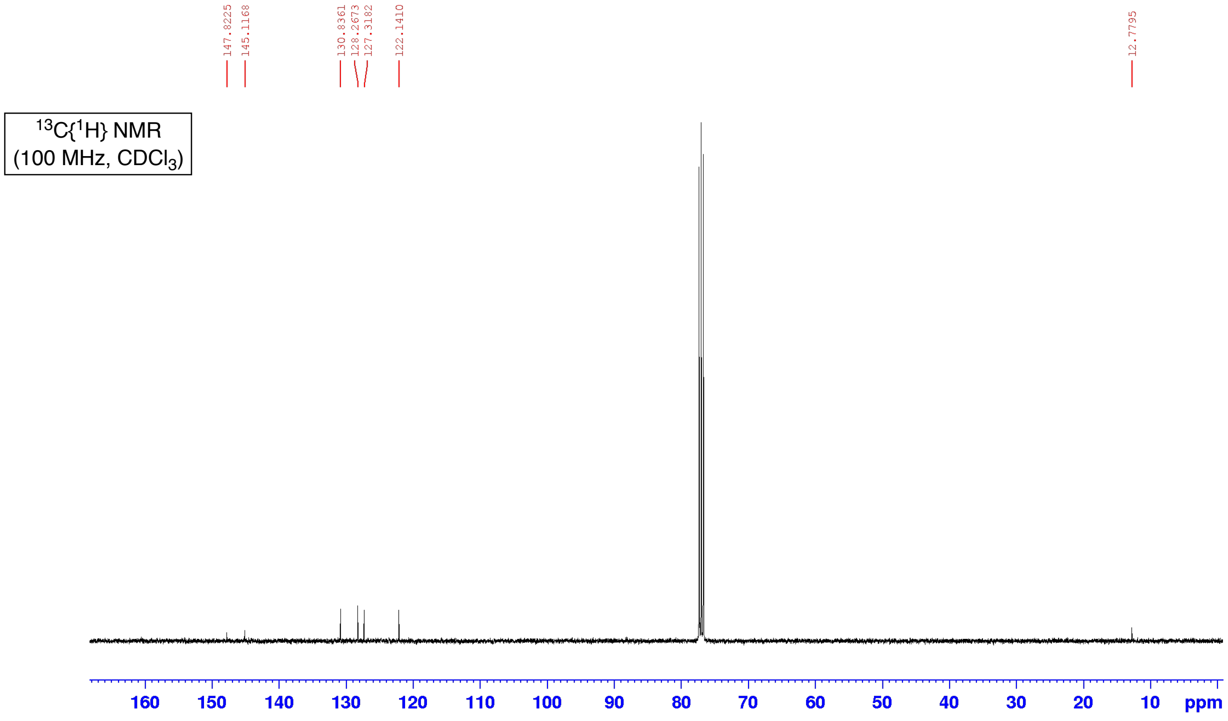

[^{1}H and ^{13}C{^{1}H} NMR Spectra of **6b**]

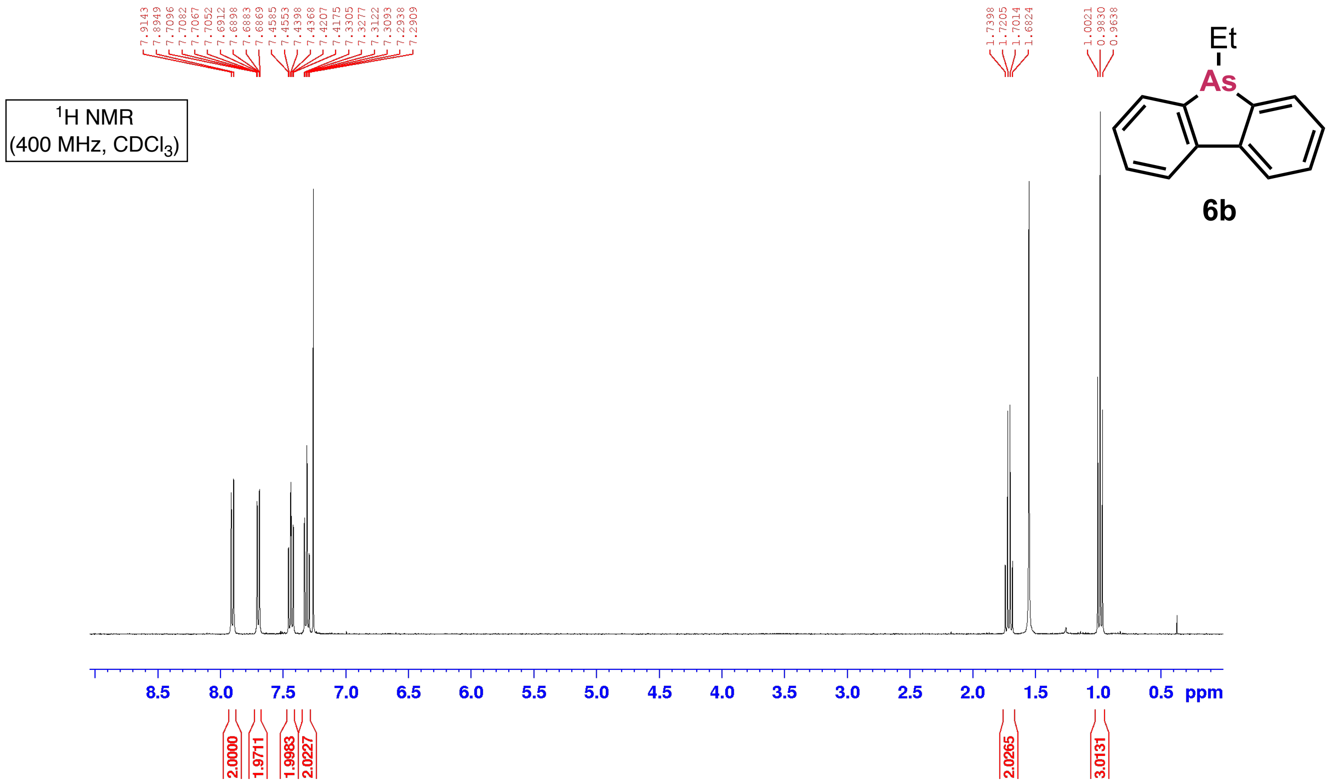

6b

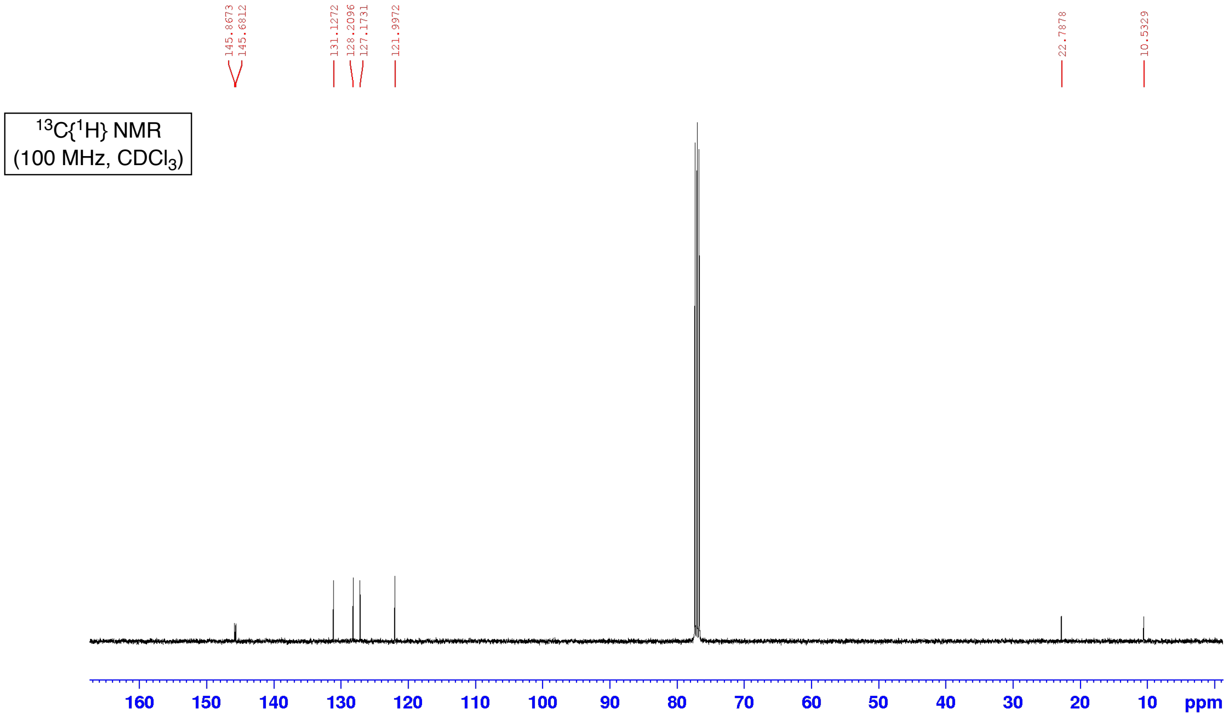

Supplement: SC-016-D5SC05528H-s001 [file SC-016-D5SC05528H-s001.pdf]
